# LIN-39 is a neuron-specific developmental determinant of longevity in *Caenorhabditis elegans* with reduced insulin signaling

**DOI:** 10.1038/s41467-025-61786-y

**Published:** 2025-07-16

**Authors:** Alan Kavšek, Jérôme Salignon, Lluís Millan-Ariño, Patryk Marcinkowski, Ilke Sen, Christian G. Riedel

**Affiliations:** 1 Integrated Cardio Metabolic Centre (ICMC) and Division of Biosciences and Nutrition, Department of Medicine, Karolinska Institute, Huddinge, Sweden; 2 Faculty of Life Sciences: Food, Nutrition, and Health, Chair of Nutritional Physiology, University of Bayreuth, Kulmbach, Germany; 3 Department of Molecular Biosciences, The Wenner-Gren Institute, Stockholm University, Stockholm, Sweden; 4 Present Address: Faculty of Medicine, Mondor Institute for Biomedical Research, INSERM U955, Université Paris Est Créteil, Créteil, France

**Keywords:** Chromatin remodelling, Transcriptional regulatory elements, Neurogenesis

## Abstract

The nuclear chromatin landscape changes with age. Here, we investigate whether chromatin alterations distinguish also animals with unusual aging rates, focusing on *Caenorhabditis elegans* with reduced insulin/IGF-like signaling (IIS), i.e., *daf-2* mutants. In these animals, enhancer regions that close with age tend to open and become transcriptionally active. We identify LIN-39 as a transcription factor (TF) binding these regions and being required for the longevity of *daf-2* mutants. LIN-39 acts during late development in hermaphrodite-specific VC motor neurons – at a time when these undergo maturation. LIN-39-mediated longevity requires DAF-16/FOXO, suggesting cooperation of both TFs in VC neurons to open enhancers. Our findings argue that longevity of *daf-2* mutant hermaphrodites relies on a signal emitted by properly matured VC neurons, and due to its essential role in this maturation process LIN-39 becomes a rare example of a development-specific lifespan determinant.

## Abstract (web summary)

Kavšek et al. reveal in this *C. elegans* study that the LIN-39 and DAF-16 transcription factors cooperate to regulate enhancer activity in VC neurons and ensure proper neuron maturation. This in turn is required for longevity under reduced IIS conditions, linking chromatin dynamics to lifespan extension.

## Introduction

Aging has become the greatest risk factor for human mortality and the occurrence of many chronic non-communicable diseases. Thus, intense search is underway to identify interventions that slow or even revert aging and thereby help humans lead longer and healthier lives. Multiple pathways have already been shown to modulate aging and ultimately lifespan across species, offering compelling leads for the identification of such interventions^1–3^.

One of the most potent aging-regulatory pathways known to date is the nutrient-dependent insulin/IGF-like signaling (IIS) pathway. A reduction in IIS leads to increased stress resistance, a reduced rate of aging, and ultimately to longevity across metazoans^4–6^. Consistently, targeting IIS has been found to defer age-related morbidities like metabolic diseases, cardiovascular diseases, or Alzheimer’s disease in model organisms^7–9^. The mechanisms by which reduced IIS exerts these benefits have been intensely studied for the last two decades. Central to these mechanisms is a transcription factor (TF) called DAF-16/FOXO, which is activated by reduced IIS and in turn drives the expression of a wide panel of stress resistance and longevity promoting genes. However, many other TFs, such as SKN-1^10^, HLH-30^11^, HSF-1^12^, and their co-factors and regulators are also involved, leaving the overall picture far from complete (for reviews see refs. ^13–16^).

During aging in humans, numerous phenotypes arise that influence the aging process and organismal health, commonly referred to as the hallmarks of aging^1^. One of these hallmarks refers to epigenetic changes in the chromatin landscape – a phenomenon that has been observed across eukaryotes^17^. While the exact relation between these changes and aging remains unclear, studies in numerous species have shown that perturbations of the epigenome can be sufficient to change the rate of aging. For instance, in *C. elegans*, loss-of-function mutations in the enzymes that add or remove H3K4me3 marks – the COMPASS complex and RBR-2 – extend or shorten the animals’ lifespan, respectively^18,19^. Similarly, overexpression of the histone deacetylases Sir2 in yeast or SIRT6 in mice extends the lifespan of these species^20,21^. This implies that aging may be driven at least partially by changes in the chromatin landscape^18,22^.

These observations prompted us to ask the following questions, using the model organism *C. elegans*: Given that the chromatin landscape changes with age in wild-type animals^23^, are slow-aging and hence long-lived IIS mutants characterized by a chromatin landscape that is different from the one of wild-type animals? And if so, are such differences contributing to the longevity of these animals? During previous work, we found initial support for this hypothesis, namely by showing that the chromatin remodeler SWI/SNF is required to achieve longevity under reduced IIS^24^. However, no systematic approach to chart the chromatin changes and their consequences in IIS mutants has been undertaken so far.

Here, we filled this gap by generating and integrating ATAC-seq and mRNA-seq data of aging wild-type *C. elegans* and of long-lived *C. elegans* with reduced IIS. Strikingly, we found that enhancer regions were becoming both less accessible and less transcribed during wild-type aging, and that this pattern was reversed under reduced IIS. These regions were enriched for the binding sites of several TFs, one of which was the homeodomain-containing TF (also referred to as HOX gene) *lin-39*. Following this lead, we discovered that *lin-39* is required for the longevity of animals with reduced IIS, through a role that it fulfills during development of the nervous system – specifically during the maturation of VC neurons – and that involves the well-established aging-preventive TF DAF-16/FOXO. Collectively, these findings reveal a mechanism in which LIN-39 functions as a developmental determinant in the nervous system, equipping VC neurons with critical properties that promote lifespan extension under reduced IIS.

## Results

### Aging *C. elegans* undergo chromatin accessibility changes

**Fig. 1:**
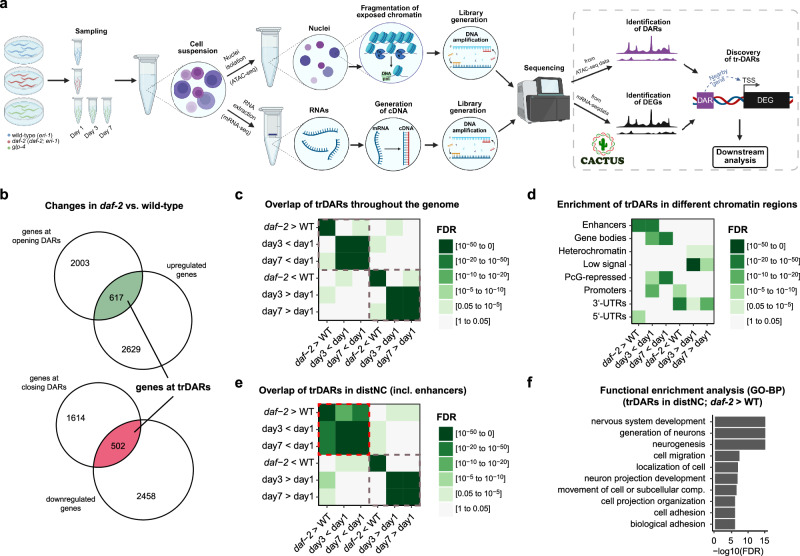
Identification of transcriptionally relevant chromatin accessibility changes that arise during aging in wild-type animals and in animals with reduced insulin/IGF-like signaling (IIS). **a** Workflow. ATAC-seq and mRNA-seq experiments were conducted on *eri-1(mg366)*, *daf-2(e1370); eri-1(mg366)*, and *glp-4(bn2) C. elegans* at the indicated timepoints. Data preprocessing and downstream analyses were performed with the pipeline Cactus^25^. The ATAC-seq data revealed differentially accessible regions (DARs), while the mRNA-seq data revealed differentially expressed genes (DEGs) between the investigated conditions. Eventually, both datasets were combined to identify DARs causing correlating gene expression changes at their closest genes (i.e. DARs that open and where the closest gene is upregulated or DARs that close and where the closest gene is repressed) (trDARs). Created in BioRender. Riedel, C. (2025) https://BioRender.com/iqifhdy. **b** Venn diagrams showing overlaps between DEGs and the genes proximal to DARs. **c** Heatmap showing the overlap of trDARs throughout the genome between the indicated conditions. Note: “x > y” refers to regions that are open in x and closed in y, while “x <y” refers to regions that are closed in x and open in y. **d** Heatmap showing the enrichment of all trDARs in different chromatin states, adapted from ref. ^26^. Note: “x > y” refers to regions that are open in x and closed in y, while “x < y" refers to regions that are closed in x and open in y. **e** Heatmap showing the overlap of distNC-localized trDARs between the indicated conditions. Note: “x > y” refers to regions that are open in x and closed in y, while “x < y” refers to regions that are closed in x and open in y. **f** Barplot showing a functional enrichment analysis of the genes proximal to distNC-localized trDARs that open under reduced IIS. Some terms were adapted or shortened for improved readability (see Supplementary Table S7 for the original terms). trDARs transcriptionally-relevant differentially accessible regions, day1, day3, and day7: age of the adult *glp-4* mutants, WT: *eri-1(mg366)*, *daf-2: daf-2*(*e1370*)*; eri-1(mg366)*, *glp-4: glp-4(bn2)*, distNC distal non-coding, PcG Polycomb Group, FDR False Discovery Rate, GO-BP Gene Ontology-Biological Processes. Source data are provided as a Source Data file.

We began by determining the chromatin accessibility and gene expression changes that occur during normal aging in the soma of wild-type *C. elegans*. To this end, we used germline-deficient *glp-4* mutant animals and conducted ATAC-seq and mRNA-seq on samples from day 1, 3, and 7 of adulthood (Fig. 1a). Chromatin accessibility changes and transcriptional changes in proximal genes were defined using a recently developed analysis pipeline called Cactus, which facilitates data preprocessing, differential and enrichment analysis, and the combination of omics modalities (Fig. 1a)^25^. First, we determined which genomic regions either opened or closed with age. Consistent with chromatin changes being a hallmark of aging, we observed abundant chromatin reorganization, identifying 9196 differentially accessible regions (DARs) that had opened by day 3 and 7007 that had opened by day 7 when compared to day 1 of adulthood (Supplementary Table S1). In addition, we identified 9968 DARs that had closed by day 3 and 7171 that had closed by day 7 when compared to day 1 of adulthood (Supplementary Table S1). Many of these DARs also showed correlating transcriptional changes of their neighboring genes, with 3258 DARs that had opened by day 3 and 2427 that had opened by day 7 leading to induction of their closest genes, in addition to 3460 DARs that had closed by day 3 and 2839 that had closed by day 7 leading to repression of their closest genes (Supplementary Tables S2, S3). For the remainder of this study, we refer to these as transcriptionally relevant DARs (trDARs). Most (~55%) of the trDARs that existed on day 7 had already started to emerge by day 3 (Supplementary Table S3), consistent with a wide number of functionally relevant chromatin accessibility changes arising and then presumably persisting with age.

### Reducing IIS causes broad chromatin accessibility changes

Having confirmed this hallmark of aging in wild-type animals, we then wondered if animals with an altered rate of aging, such as slow-aging and thus long-lived animals, would distinguish themselves by an altered chromatin landscape, too. We used *eri-1(mg366)* background animals that offer enhanced RNA interference (RNAi) and hence would be useful during downstream analyses. In this background, we then compared otherwise wild-type and long-lived *daf-2(e1370)* animals, the latter of which have reduced IIS, by performing ATAC- and mRNA-seq on day 1 of adulthood (Fig. 1a). Intriguingly, we observed substantial *daf-2(e1370)*-induced chromatin accessibility changes: 3912 DARs had opened and 2850 DARs had closed in these mutants (Supplementary Table S1). Among these, there were 1052 trDARs that activated a total of 617 unique proximal genes and 734 trDARs that repressed a total of 502 unique proximal genes (Fig. 1b, Supplementary Tables S2, S3). We conclude that just how changes in the chromatin landscape are a hallmark of normal aging, they are also a major distinguishing feature of long-lived animals with reduced IIS and have a noticeable impact on gene expression.

### Numerous enhancers close with age but open under reduced IIS

One can hypothesize that longevity is achieved by preventing molecular changes that occur during aging in wild-type animals. Following this idea, genomic regions that close and become repressed or that open and become activated with age might experience opposite changes under reduced IIS, which in turn might contribute to these animals’ longevity. First, we looked for such events across the genome, but the overlap between trDARs affected by reduced IIS and trDARs that change in the opposite direction during normal aging was low (Fig. 1c, Supplementary Table S3).

Interestingly though, this changed when limiting the analysis to specific regions of the genome. Using an epigenetic-marks-based chromatin state map^26^, we found two regions highly enriched for trDARs changing in opposite directions during normal aging and reduced IIS, namely enhancers and 3’-UTRs (Fig. 1d, Supplementary Table S4). We then limited our analysis to these two regions. However, we used a more customized conventional genome annotation and expanded our view on enhancers to now span the entire intergenic non-coding space (distNC – distal non-coding)^27^. Specifically, for the distNC regions, we detected a strong and significant overlap between trDARs that open in long-lived *daf-2* mutants and trDARs that close during normal aging (Fig. 1e, Supplementary Table S5). Substantially lower overlap was observed for trDARs that close in long-lived *daf-2* mutants and trDARs that open during normal aging in the distNC regions (Fig. 1e, Supplementary Table S5). Likewise, overlaps between trDARs in 3’-UTRs occurring during normal aging and trDARs in 3’-UTRs occurring in opposite direction in *daf-2* mutants were low (Supplementary Fig. S1a, Supplementary Table S6). These results suggested that distNC regions could play a particular role in the regulation of aging, where keeping specific sites open might slow down aging and thus extend the animals’ lifespan. Our results from Fig. 1d implied that these are enhancer regions. Cross-referencing with the epigenetic-marks-based map from ref. ^26^ confirmed this assumption (Supplementary Fig. S1b).

To identify the cellular processes regulated by these enhancer-localized trDARs in *daf-2* mutants, we conducted functional enrichment analysis on their closest neighboring genes (Fig. 1f, Supplementary Table S7). The strongest enrichment was observed for genes involved in the development of the nervous system. Overall, our results indicate that reduced IIS counteracts the closure of enhancer regions and resulting gene repression occurring during aging in wild-type animals, and that this may have functional relevance in the developing nervous system.

### LIN-39 binds opening enhancers and promotes longevity

**Fig. 2:**
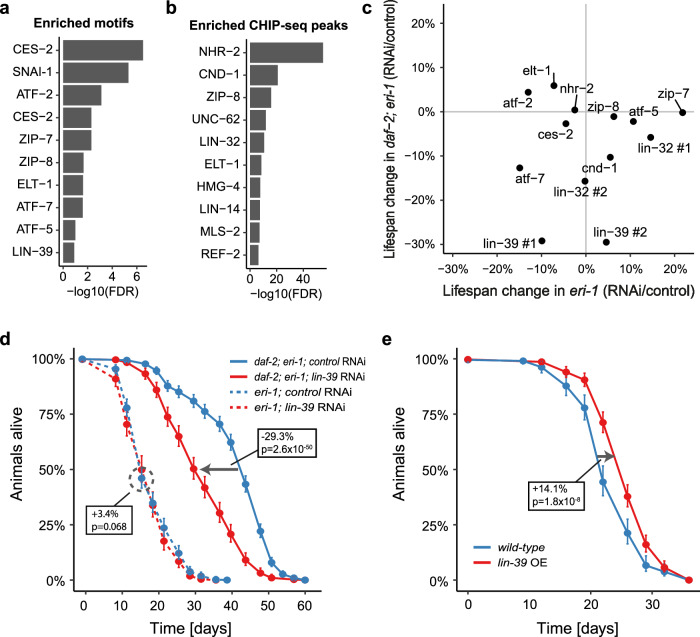
Screening of transcription factors (TFs) that are predicted to bind distNC-localized trDARs opening under reduced IIS reveals LIN-39 as a driver of longevity. **a** The ten most significant enrichments of TF DNA-binding motifs from the Cis-BP database (build 2.00) in trDARs that open under reduced IIS. **b** The ten most significant enrichments of TF ChIP-seq peaks from modENCODE in trDARs that open under reduced IIS. **c** Screen for lifespan phenotypes of selected candidates from (**a**) and (**b**). Candidate TFs were knocked down by RNAi from the L1 stage in *eri-1(mg366)* and *daf-2(e1370); eri-1(mg366)* mutant *C. elegans*. “#1” and “#2” denote distinct RNAi clones targeting the same respective gene. **d** Kaplan–Meier survival estimates with 95% confidence intervals for *eri-1(mg366)* and *daf-2(e1370); eri-1(mg366)* mutant *C. elegans* grown from the L1 stage on the indicated RNAi bacteria. (*n* = 445 (*eri-1*; control RNAi), 272 (*eri-1*; *lin-39* RNAi), 720 (*daf-2*; *eri-1*; control RNAi), 409 (*daf-2*; *eri-1*; *lin-39* RNAi); for results from a second independent biological replicate experiment, see Supplementary Table S10). **e** Kaplan–Meier survival estimates with 95% confidence intervals for wild-type (N2) *C. elegans* and the same animals carrying an overexpression construct of LIN-39. (*n* = 211 (wild-type), 367 (*lin-39* OE); for results from a second independent biological replicate experiment, see Supplementary Table S10). Boxes state changes in median lifespan and the associated *p*-values (based on two-sided log-rank tests) for the indicated comparisons. OE overexpression. Source data are provided as a Source Data file.

Next, we wanted to identify molecular factors that cause or utilize this increase in chromatin accessibility at enhancer regions under reduced IIS. It is conceivable that specific TFs can bind to these regions and promote lifespan-extending gene expression changes. Therefore, we conducted enrichment analyses for TF binding based on TF DNA-binding motifs from the Cis-BP database^27,28^ and ChIP-seq peaks generated by the modENCODE consortium^29^. Surveying a total of 420 TFs, we found 19 TFs whose motifs or binding sites were enriched in the opening enhancer-localized trDARs (Fig. 2a, b, Supplementary Tables S8, S9). To test if some of these TFs are required for the longevity of *daf-2* mutants, we determined their loss-of-function phenotypes. Staying in the *eri-1(mg366)* background used already in Fig. 1, we now performed lifespan assays in which we knocked down the TF candidates in otherwise wild-type or *daf-2(e1370)* mutant animals from the L1 stage onwards. Some candidates were excluded because their loss was annotated to cause lethality or impair the development of *C. elegans*, or because we could not obtain suitable RNAi clones from the commercially available genome-wide RNAi libraries. This experiment resulted in one standout candidate which was the homeodomain-containing TF LIN-39 (Fig. 2c, Supplementary Fig. S2, Supplementary Table S10). Knockdown of this TF caused the strongest lifespan reduction in *daf-2* mutant animals among all the tested candidates, while it only caused a marginal lifespan change in wild-type animals (Fig. 2c, d, Supplementary Table S10). These results indicate that LIN-39 is specifically required for animals with reduced IIS to become long-lived.

We have shown that trDARs in distNC regions which open in *daf-2* mutants tend to close during normal aging in wild-type animals. Hence, we wondered if the lifespan-extending activities of LIN-39 were actually compromised in wild-type animals and whether this could be overcome by overexpressing LIN-39. Indeed, this was the case (Fig. 2e, Supplementary Table S10), arguing that LIN-39 is in itself a longevity-promoting TF.

### LIN-39 affects lifespan through a function in the nervous system

**Fig. 3:**
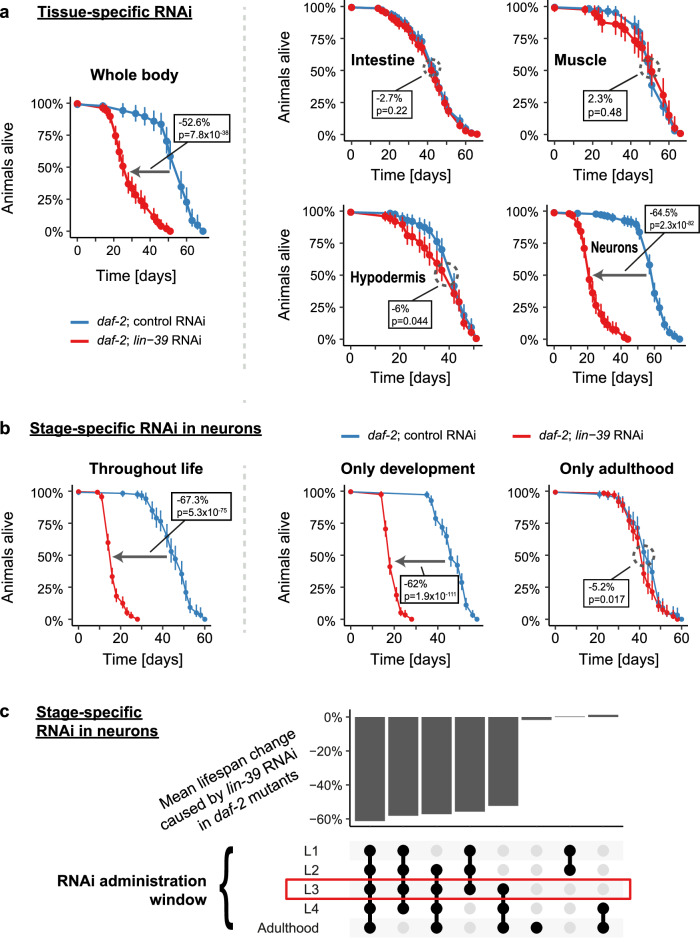
LIN-39 promotes longevity under reduced IIS from the nervous system and exclusively during later larval development. **a** Kaplan–Meier survival estimates with 95% confidence intervals for *daf-2(e1370)* mutant *C. elegans* with functional RNAi in either the whole body or the indicated tissues, grown on the indicated RNAi bacteria. (*n* = 83 (whole body; control RNAi), 151 (whole body; *lin-39* RNAi), 125 (hypodermis; control RNAi), 124 (hypodermis; *lin-39* RNAi), 291 (intestine; control RNAi), 264 (intestine; *lin-39* RNAi), 94 (muscle; control RNAi), 69 (muscle; *lin-39* RNAi), 174 (neurons; control RNAi), 142 (neurons; *lin-39* RNAi); for results from a second independent biological replicate experiment, see Supplementary Table S11). **b** Kaplan–Meier survival estimates with 95% confidence intervals for *daf-2(e1370); sid-1(pk3321)* mutant *C. elegan*s, in which *sid-1* was reconstituted only in the nervous system, resulting in neuron-specific RNAi. Animals were grown on the indicated RNAi bacteria either throughout life, exclusively during development (and then shifted to *dcr-1* RNAi during adulthood to inactivate the RNAi), or exclusively during adulthood (after having been grown on HT115 bacteria during development). (*n* = 118 (throughout life; control RNAi), 248 (throughout life; *lin-39* RNAi), 215 (only development; control RNAi), 233 (only development; *lin-39* RNAi), 121 (only adulthood; control RNAi), 134 (only adulthood; *lin-39* RNAi); for independent replication of these findings, see Fig. 3c and Supplementary Table S12). **c** Same experimental setup as under (**b**), but more knockdown-windows were tested. Results are displayed as an UpSet plot showing the changes in median lifespan induced by neuron-specific *lin-39* knockdown. For conditions tested in multiple experiments, percentages were averaged. These included development (3 independent experiments), adulthood (2 independent experiments), and throughout life (2 independent experiments). The actual survival curves are shown in (**b**) and Supplementary Fig. S4b. (for *n*, see Supplementary Table S12; the experiment in conjunction with Fig. 3b provides internal biological replication of the key findings due to overlapping or matching knockdown windows). Boxes state changes in median lifespan and the associated *p*-values (based on two-sided log-rank tests) for the indicated comparisons. Source data are provided as a Source Data file.

Since LIN-39 is required for the longevity of *daf-2* mutants, we wondered in which tissue LIN-39 confers this role. To assess this, we conducted lifespan assays in animals where *lin-39* was knocked down only in specific tissues. Specifically, we used *daf-2* mutant *C. elegans* strains that were deficient for RNAi due to mutation of either *rde-1* or *sid-1*, and in which the expression of these genes was restored in only a single tissue of interest^30,31^. By this approach, we tested the relevance of *lin-39* in the hypodermis, muscle, intestine, and nervous system. Strikingly, knockdown of *lin-39* in the nervous system fully phenocopied the effect of a whole-body knockdown of *lin-39*, while no such phenotype was observed from knockdown of *lin-39* in the other tested tissues (Fig. 3a, Supplementary Table S11). The lifespan decrease caused by neuron-specific *lin-39* RNAi was even more substantial than that obtained from whole-body RNAi, shortening the lifespan of *daf-2* mutants by ~65% and 53%, respectively (Fig. 3a, Supplementary Table S11). This is likely due to the neuronally reconstituted *sid-1* being expressed at higher than physiological levels, which should enhance neuronal *lin-39* knockdown^30^. Taken together, these results show that LIN-39 promotes longevity under reduced IIS in a tissue-specific manner, primarily through a function in neurons.

### LIN-39 regulates organismal lifespan during development

LIN-39 has already been a subject of study for over three decades, during which multiple functions were identified. Most notably, it is important during development, being involved in body patterning and in establishment of the nervous system by regulating the migration of QR neuroblast descendants^32^; but it also plays a post-developmental role, whereby it maintains the identity of motor neurons throughout adulthood^33^. Given such developmental and post-developmental functions in the nervous system, we wondered at what stage LIN-39 promotes longevity. To address this, we knocked down *lin-39* specifically in neurons, either exclusively during development, during adulthood, or throughout life, and evaluated the resulting impact on *daf-2* mutants’ lifespan. Remarkably, knockdown of *lin-39* only during development suppressed the longevity of *daf-2* mutants almost to the same extent as knockdown of *lin-39* throughout life (Fig. 3b, Supplementary Table S12). On the other hand, knockdown of *lin-39* only during adulthood had almost no effect on the lifespan of *daf-2* mutant animals.

To further narrow down the time window during which LIN-39 regulates longevity, we then performed stage-specific knockdown of *lin-39* only during parts of development. Interestingly, *lin-39* RNAi impaired longevity only when animals were exposed to RNAi bacteria at the L3 stage, with no effect observed when exposure occurred exclusively at other developmental or adult stages (Fig. 3c, Supplementary Fig. S4b, Supplementary Table S12). As a control, we carried out stage-specific RNAi of *daf-16*, as a known means to suppress the longevity of *daf-2* mutant animals also during adulthood^34^. *daf-16* RNAi exhibited a longevity-suppressing effect at all stages of life (Supplementary Fig. S4a). Even though stage-specific RNAi has a certain degree of imprecision, as it may take several hours to take effect and also hours to fully subside again after the knockdown has been stopped, our findings argue that LIN-39 promotes the longevity of *daf-2* mutants by acting in the developing nervous system, through processes that occur around the L3 stage. Furthermore, the dispensability of LIN-39 at later stages implies that its role lies in the birth or maturation of neurons, rather than in their maintenance – providing a prime example of a developmental determinant of the aging process.

### LIN-39 opens enhancers of age-regulated neuronal genes

It has long been known that the nervous system can modulate the organism’s rate of aging. Nevertheless, the underlying mechanisms are diverse^35,36^, raising the question of how neuronal LIN-39 achieves its longevity-promoting role in *daf-2* mutant *C. elegans*. To better understand the mechanistic consequences of *lin-39* depletion, we conducted ATAC-seq in *daf-2* mutants in the presence or absence of *lin-39* RNAi. We found that LIN-39 promotes opening and closing of chromatin in 303 and 331 regions, respectively (Supplementary Table S13). Strikingly, ~70% of regions that close upon *lin-39* knockdown in *daf-2* mutants (i.e., 212 out of 303) overlap with regions that open under reduced IIS (Supplementary Fig. S3a, Supplementary Table S13). This finding strongly supports the view that LIN-39 not only binds such regions but also contributes to some of the chromatin accessibility changes that occur under reduced IIS. DARs opened by LIN-39 were predominantly enriched for enhancers (Supplementary Fig. S3b, Supplementary Table S14), which is consistent with our previous observation that LIN-39 binding sites are enriched within enhancers that open under reduced IIS (Fig. 2a).

**Fig. 4:**
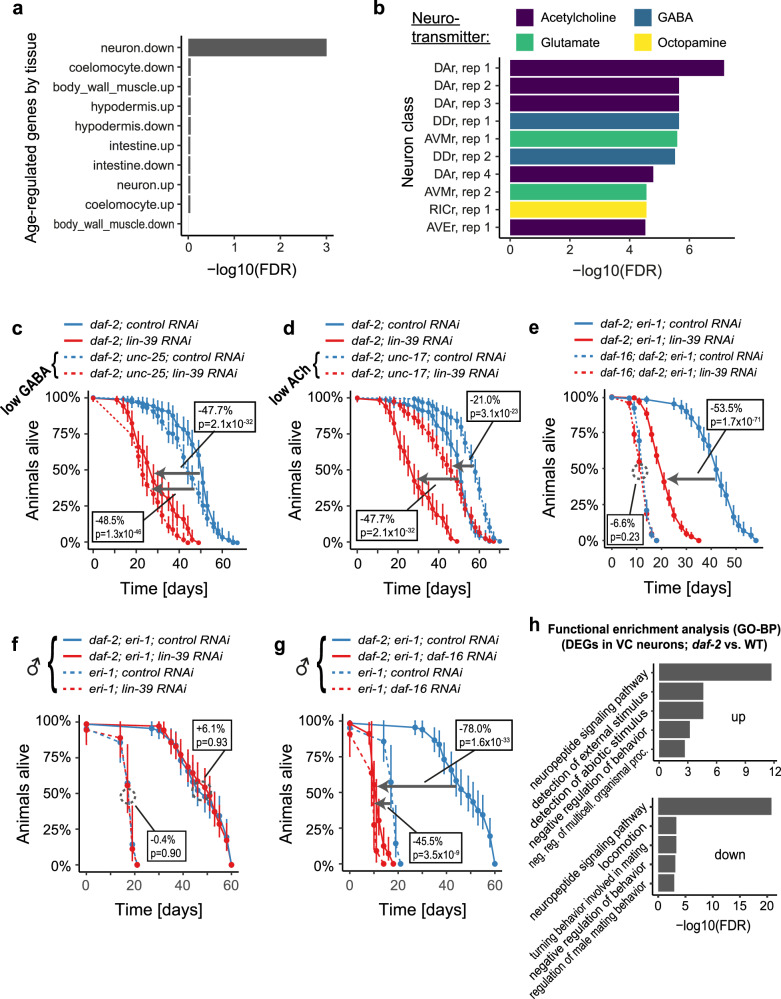
LIN-39 promotes longevity under reduced IIS in a manner dependent on cholinergic VC neurons and the TF DAF-16/FOXO. **a**, **b** distNC-localized DARs opened by LIN-39 in *daf-2(e1370)* mutants were assigned to their closest neighboring gene, resulting in a list of genes predicted to be regulated by LIN-39. **a** A barplot showing the enrichment of these LIN-39-regulated genes in genes differentially regulated with age in various tissues, as reported in ref. ^37^. **b** A barplot showing the enrichment of these LIN-39-regulated genes in genes expressed in various *C. elegans* neuron classes, as reported in refs. ^38,39^. **c**, **d** Kaplan–Meier survival estimates with 95% confidence intervals for *daf-2* mutants deficient in GABA signaling (*daf-2(e1370); unc-25(e156*) animals) (**c**) or ACh signaling (*daf-2(e1370)*; *unc-17(e245)* animals) (**d**) grown from the L1 stage on the indicated RNAi bacteria. (*n* = 106 (*daf-2*; control RNAi), 92 (*daf-2*; *lin-39* RNAi), 156 (*daf-2*; *unc-25*; control RNAi), 143 (*daf-2*; *unc-25*; *lin-39* RNAi), 163 (*daf-2*; *unc-17*; control RNAi), 195 (*daf-2*; *unc-17*; *lin-39* RNAi); for results from a second independent biological replicate experiment, see Supplementary Table S20). **e** Kaplan–Meier survival estimates with 95% confidence intervals for *daf-2(e1370); eri-1(mg366)* and *daf-16(mgDf47); daf-2(e1370); eri-1(mg366)* mutants grown from the L1 stage on the indicated RNAi bacteria. (*n* = 133 (*daf-2*; control RNAi), 291 (*daf-2*; *lin-39* RNAi), 171 (*daf-2*; *daf-16*; control RNAi), 193 (*daf-2*; *daf-16*; *lin-39* RNAi); for results from a second independent biological replicate experiment, see Supplementary Table S21). **f**, **g** Kaplan–Meier survival estimates with 95% confidence intervals for *eri-1(mg366) and daf-2(e1370); eri-1(mg366)* mutant male animals grown from the L1 stage on the indicated RNAi bacteria. (*n* = 20 (*eri-1*; control RNAi), 10 (*eri-1*; *daf-16* RNAi), 17 (*eri-1*; *lin-39* RNAi), 66 (*daf-2*; *eri-1*; control RNAi), 55 (*daf-2; eri-1*; *daf-16* RNAi), 69 (*daf-2; eri-1*; *lin-39* RNAi); results are derived from combination of two independent biological replicate experiments with consistent results). **h** Barplots showing functional enrichment analyses of the genes up- or downregulated in VC neurons under reduced IIS. Some terms were adapted or shortened for improved readability (see Supplementary Table S26 for the original terms). Boxes state changes in median lifespan and the associated *p*-values (based on two-sided log-rank tests) for the indicated comparisons. FDR False discovery rate, rep: replicate. Source data are provided as a Source Data file.

Next, we wondered about the relevance of these enhancer-localized DARs for the aging process. We assigned each of these regions to their closest gene and checked if these genes had been reported to undergo expression changes with age in specific *C. elegans* tissues^37^. Remarkably, we detected a strong overlap between genes proximal to regions opened by LIN-39 in *daf-2* mutants and genes downregulated during aging in neurons (Fig. 4a, Supplementary Table S15). Similar results were obtained when overlapping genes proximal to LIN-39-bound enhancers in wild-type (derived from modENCODE ChIP-seq data^29^) with genes regulated during aging in neurons (Supplementary Fig. S5a, Supplementary Table S16). Taken together, these results indicate that LIN-39 promotes longevity by promoting the expression of neuron-specific genes whose expression otherwise declines with age.

### Lifespan regulation by LIN-39 requires cholinergic signaling

*C. elegans* neurons are well-characterized and fall into a variety of classes. Hence, we evaluated whether enhancer-localized DARs opened by LIN-39 in *daf-2* mutants are functionally relevant for a specific neuron class. We compared the genes most proximal to these DARs with 118 neuronal cell type-specific gene expression profiles available from the CeNGEN project, which provides a transcriptional map of the *C. elegans* nervous system^38,39^. This analysis showed that the genes proximal to DARs opened by LIN-39 in *daf-2* mutants overlap most strongly with transcriptomic signatures of cholinergic and GABAergic neurons (GABA: γ-aminobutyric acid; Fig. 4b, Supplementary Table S17). Similar results were obtained when using the genes proximal to LIN-39-bound enhancers in wild-type animals (derived from modENCODE ChIP-seq data^29^) (Supplementary Fig. S5b, Supplementary Table S18). These observations suggest that numerous enhancer-localized DARs opened by LIN-39 regulate genes specific to cholinergic and GABAergic neurons, aligning well with recent reports identifying LIN-39 as a terminal selector for these neuron classes^33,40^.

This led us to investigate whether the activity of these specific neurons is required for the longevity-promoting role of LIN-39. To that end, we tested whether *daf-2* mutant animals with reduced acetylcholine (Ach) or GABA signaling (harboring *unc-17(e245)* or *unc-25(e156)* mutations, respectively) would still exhibit lifespan-shortening in response to *lin-39* RNAi. Loss of GABA signaling did not alter this lifespan-shortening effect: *lin-39* RNAi shortened the lifespan of *daf-2* mutants by 47.7% and that of *daf-2; unc-25* mutants by 48.5% (Fig. 4c, Supplementary Table S20). In contrast, loss of cholinergic signaling partially suppressed the lifespan-shortening effect (by 56%): *lin-39* RNAi shortened the lifespan of *daf-2* mutants by 47.7% and that of *daf-2; unc-17* only by 21% (Fig. 4d, Supplementary Table S20). Collectively, these findings reveal that LIN-39 promotes longevity in animals with reduced IIS through a function in the cholinergic nervous system.

### Lifespan regulation by LIN-39 occurs through VC neurons

Intrigued by LIN-39’s role in the cholinergic nervous system, we next sought to identify the specific subset of cholinergic neurons through which LIN-39 acts in *daf-2* mutants. To begin, we examined whether reduced IIS and/or *lin-39* loss led to any obvious substantial changes in the cholinergic nervous system. We monitored the expression of *unc-17*::GFP, a marker of cholinergic neurons, in both wild-type and *daf-2* mutant animals in the presence or absence of LIN-39. Analyses at late larval stages and day 1 of adulthood did not reveal any such obvious phenotype (Supplementary Fig. S6), implying that LIN-39 may not be required for the presence of cholinergic neurons but rather alter their characteristics. Additionally, we assessed the expression of LIN-39 in both wild-type and *daf-2* mutant animals but found no notable differences in either expression levels or spatial pattern within the nervous system (Supplementary Fig. S7). There seems to occur an upregulation of LIN-39 expression in vulval precursor cells of *daf-2* mutant animals during the L2 and L3 stages, but this result is likely not relevant for this study, given that *lin-39* RNAi in neurons is sufficient to impair the longevity of animals with reduced IIS.

Previous studies have identified several cholinergic neuron classes that express LIN-39, namely the interneurons AIY, SDQ, as well as the motor neurons DA, DB, VA, VB, AS, and VC^41,42^. In all of these neurons, LIN-39 functions as a terminal selector, meaning that its absence during birth or maturation of these neurons would disturb their identity and function. In our effort to pinpoint the specific neuron class that LIN-39 is affecting to confer longevity under reduced IIS, we then considered the fact that different neuron classes are generated and mature at distinct stages of development. Among the aforementioned LIN-39-expressing cholinergic neurons, AIY, SDQ, DA, DB, VA, VB, and AS are all born and mature early, during embryogenesis or the L1 stage. The only exception is the VC neurons, which are born at the L1 stage but continue to send out processes during the L3 and L4 stages before reaching their final mature state and function^41,43^. Furthermore, we found a significant enrichment of genes proximal to DARs opened by LIN-39 in *daf-2* mutants within transcriptomic signatures specific to VC neurons (Supplementary Table S17, ranks 16, 18, 20, 24, 33, and 44 out of 138 entries), suggesting that these neurons undergo important LIN-39-induced chromatin accessibility changes that could alter their transcriptome and ultimately their characteristics. Altogether, these observations point towards VC neurons as the neuron class through which LIN-39 promotes longevity under reduced IIS.

To test this hypothesis, we leveraged the fact that VC neurons are sex-specific, being present only in hermaphrodites while being absent from male *C. elegans*. We thus exposed *eri-1* and *eri-1; daf-2* males to control, *lin-39*, or *daf-16* RNAi and measured their lifespans. Strikingly, while both *lin-39* and *daf-16* RNAi reduced the lifespan of *daf-2* mutant hermaphrodites (Fig. 2d and Supplementary Fig. S4a), only *daf-16* RNAi affected the lifespan of *daf-2* males, while *lin-39* RNAi had no effect (Fig. 4f–g, Supplementary Table S21). This result provides strong support for the hypothesis that LIN-39 regulates longevity specifically through actions in VC neurons.

It is important to note that in most of the LIN-39-expressing cholinergic neuron classes, LIN-39 does not act as the sole terminal selector; instead, additional TFs are required to establish neuronal identity. Thus, to complement our findings and rule out the involvement of other LIN-39-expressing cholinergic neuron classes besides VC, we performed RNAi-mediated knockdown of five of these terminal selectors – cfi-1, egl-5, mab-5, unc-3, and unc-4 – in *eri-1* and *daf-2; eri-1* mutant animals. This allowed us to disturb the identity of DA neurons (through *egl-5*, *mab-5*, *unc-4*, and *cfi-1*), DB neurons (through *cfi-1*), VA neurons (through *egl-5*, *mab-5*, *unc-4*, *unc-3*, and *cfi-1*), VB neurons (through *unc-3* and *cfi-1*), and AS neurons (through *mab-5* and *unc-3*)^41^. Notably, none of these terminal selector knockdowns selectively impaired the longevity of *daf-2* mutant animals (Supplementary Fig. S5c, Supplementary Table S19), suggesting that these other neuron classes are negligible for the LIN-39-dependent phenotype described in this study.

Taken together, our data support a model in which LIN-39 promotes longevity under reduced IIS by ensuring the proper maturation of VC neurons, which in turn appear to mediate the longevity phenotype. To explore this idea further, we analyzed existing single-cell transcriptomic data from the *C. elegans* nervous system that compared wild-type and *daf-2* mutant animals^44^. Focusing specifically on the VC neurons, we identified differentially expressed genes between the two genotypes and conducted functional enrichment analysis. Notably, the most enriched term for both genes upregulated and downregulated in *daf-2* mutant animals was ‘neuropeptide signaling’ (Fig. 4h, Supplementary Table S26), suggesting that the pro-longevity effect of VC neurons in *daf-2* mutants may be mediated through the release of specific neuropeptides.

### Lifespan regulation by LIN-39 depends on the TF DAF-16

As mentioned previously, one of the key players in relaying reduced IIS into longevity-promoting outcomes is the conserved TF DAF-16^4,45^. It confers this lifespan-regulatory role predominantly in two tissues: the nervous system and the intestine^46^. Given that LIN-39 also regulates lifespan through a function in the nervous system, we tested for a genetic interaction between *daf-16* and *lin-39* in *daf-2* mutant animals. Interestingly, we found that a null mutation in *daf-16* fully suppressed the lifespan-shortening effect of *lin-39* RNAi in *daf-2* mutant animals (Fig. 4e, Supplementary Table S21), demonstrating that LIN-39 and DAF-16 act in the same aging-regulatory pathway.

FOX (Forkhead box) TFs have a tendency to act as pioneer TFs, meaning that they possess the ability to open previously closed and thus silent chromatin, e.g., by recruiting chromatin remodelers^24,47,48^. However, while FOXA is a well-established pioneer TF^49^, the pioneering capabilities of FOXO TFs like DAF-16 are less pronounced, sometimes requiring pre-existing chromatin marks or cooperation with other TFs to activate gene expression^11,48,50^. One example of such synergistic TF is HLH-30, which is essential for DAF-16 to confer longevity^11^. Our observations now argue that LIN-39 may be another TF that cooperates with DAF-16 in chromatin opening and gene activation. In fact, HOX TFs are thought to have pioneering activity themselves^51^, further underscoring this possibility.

To test this, we knocked down *daf-16* in *daf-2* mutant animals and performed ATAC-seq, identifying 5,711 DARs that are opened and 2,261 DARs that are closed by DAF-16 (Supplementary Table S22). Remarkably, as many as 65% of DARs opened by LIN-39 also required DAF-16 for their opening, and around 10% of DARs closed by LIN-39 also required DAF-16 for their closing (Supplementary Fig. S8a, Supplementary Table S22). Inspection of these regions revealed that while DAF-16-opened DARs were enriched for both promoters and enhancers (Supplementary Fig. S8b, Supplementary Table S23), those overlapping with LIN-39-opened DARs were only enriched for enhancers (Supplementary Fig. S8c, Supplementary Table S24). Functional enrichment analysis of these co-opened DARs attributed them, amongst others, to development and morphogenesis of the nervous system (Supplementary Fig. S8d, Supplementary Table S25). In conjunction with the previous paragraph, these results suggest a cooperation between DAF-16 and LIN-39 in facilitating the opening of specific enhancer regions, presumably in VC neurons, that drive longevity-promoting events in *daf-2* mutant animals.

## Discussion

It has been well established that aging coincides with substantial changes in the chromatin landscape, and that disturbance of this landscape can alter the rate at which aging progresses. But while this phenomenon was predominantly observed in wild-type animals, it had remained unknown whether changes in chromatin are also a distinguishing feature of animals with unusual aging rates, living exceptionally short or long. Our study addressed this question by focusing on one of the strongest longevity scenarios in metazoans – reduced IIS. First, we could show that reduction of IIS induces a plethora of chromatin accessibility changes, many of which take place in enhancers (Fig. 1b, d). Notably, enhancer regions opening and becoming transcriptionally active under reduced IIS strongly overlapped with enhancer regions closing and becoming transcriptionally repressed during aging in wild-type animals (Fig. 1e). These enhancers were enriched for the DNA-binding binding motif of the HOX TF LIN-39, which turned out to be required for reduced IIS to promote longevity (Fig. 2a, d). Taken together, our findings suggested that accessibility changes in specific enhancer regions may be important to counteract aging and associated phenotypes. This hypothesis resonates with a recent study describing a possible link between epigenetic dysregulation of enhancers in humans and the age-related morbidity of Alzheimer’s disease^52^.

Although the precise origin of the enhancer changes remains uncertain, our data supports a model in which, at least in certain neurons, these changes are driven by a cooperation between LIN-39 and DAF-16. Arguments for this model are the following: First, it is known that DAF-16 is required for the aging-preventive benefits of reduced IIS, but its activation alone is not sufficient to confer these benefits^53^. Similarly, we found that overexpression of LIN-39 in wild-type animals, where DAF-16 is mostly inactive, was sufficient to extend lifespan, but only modestly and not nearly to the extent observed under reduced IIS conditions (Fig. 2e). Both observations argue that LIN-39 and DAF-16 require additional support to exert their aging-preventive benefits – support which they may provide to each other. Second, FOXO and HOX TFs have both been described to possess pioneering activity – that is, an ability to bind and open closed and transcriptionally silent chromatin and elicit gene activation, often via recruitment of chromatin remodelers^24,51^. However, they were found to sometimes be insufficient in this role, e.g., requiring preexisting chromatin marks or other TFs^11,48,50^. Third, our ATAC-seq results revealed that LIN-39 and DAF-16 regulate overlapping sets of DARs in enhancer regions (Supplementary Fig. S8a, c). Fourth, even though DAF-16 acts also in other tissues, both DAF-16 and LIN-39 must be present in the nervous system for animals with reduced IIS to become long-lived. Finally, *daf-16* is epistatic to *lin-39* in lifespan assays (Fig. 4e), further supporting functional interaction between the two. All this evidence is consistent with the following model: 1) reduced IIS activates DAF-16 throughout the organism, including within the nervous system; 2) DAF-16 then, besides other actions, attempts to open and activate multiple enhancer regions, but requires the assistance of LIN-39 to do so effectively – at least in certain neurons including the VC motor neurons; 3) through their cooperative action, these TFs open specific enhancers and drive gene expression programs that confer VC neurons with longevity-promoting properties under reduced IIS (Supplementary Fig. S9). Critically, these events must occur during the maturation of VC neurons, around the L3 stage, and have no impact if they occur earlier or later in life. This positions LIN-39 as a developmental determinant of adult lifespan, only a few of which have been reported before^54–56^.

It needs to be clarified that despite the proposed cooperation between LIN-39 and DAF-16 in regulating enhancers in the nervous system and despite the lifespan-extending function of LIN-39 under reduced IIS being confined to the maturation of VC neurons, the lifespan-regulatory roles of IIS and DAF-16 are broader: DAF-16 and other IIS components regulate lifespan also in other tissues, most importantly in the intestine^46,57^. Further, as could be seen also in Supplementary Fig. S4a, DAF-16 is required for the longevity of *daf-2* mutant animals throughout life, and not just around the L3 stage. Finally, DAF-2 and DAF-16 influence lifespan also in males – which lack the VC neurons (Fig. 4g). This shows that reduced IIS imposes its benefits not only by controlling the characteristics of VC neurons but that its spatial distribution is more complex and that it involves yet additional mechanisms.

Coming back to LIN-39, we are left with two questions: First, how do mature VC neurons contribute to slowed aging and extended lifespan in animals with reduced IIS? Here we know that VC neurons are cholinergic motor neurons, and our gene expression analysis comparing VC neurons from wild-type and *daf-2* mutant animals revealed marked differences in neuropeptide profiles (Fig. 4h). This finding suggests that the longevity-promoting effect of VC neurons may stem from the secretion of a distinct set of neuropeptides under reduced IIS. These neuropeptides could function as endocrine signals, modulating aging-related pathways, such as stress response pathways, in distal tissues. Alternatively, they may act as neuromodulators, influencing behaviors or motor functions that contribute to enhanced survival. For example, the neuropeptide NLP-12 has been shown to influence behavioral adaptation to food availability^58^. The second question is why this mechanism exists and specifically involves VC neurons but no other neuron class. Given the observed *lin-39*-dependent lifespan phenotypes, we see its purpose in adapting the organism’s physiology to scenarios of decreased nutrient availability; and since VC neurons predominantly control egg laying-associated motor functions in the hermaphrodite^59^, they might be in the unique position to intersect nutrient-dependent signaling with signaling to or from the reproductive system. However, future studies will be required to provide final answers to these questions.

In summary, our study highlights an unprecedented interplay between a HOX TF, a FOXO TF, and a reproduction-associated class of cholinergic motor neurons, which is required for aging prevention and longevity under reduced IIS. Given that most of these components are conserved across metazoans, our findings may also have relevance for humans and thus inspire future investigation into how nutrient-dependent signaling pathways regulate aging in ourselves.

## Methods

### *C. elegans* strains and their maintenance

*C. elegans* were grown on the *Escherichia coli* strain OP-50 using standard methods^60^. For a full list of strains used in this study please see Supplementary Table S27. Experiments were conducted using hermaphrodites, unless stated otherwise.

### RNAi by feeding

RNAi was conducted by growing *C. elegans* on the *E. coli* strain HT115 containing dsRNA-expressing plasmids that target a specific gene of interest, as described in ref. ^61^. RNAi clones targeting *atf-2*, *atf-5*, *cnd-1*, *zip-8*, *elt-1*, *ces-1*, *ces-2*, *mab-5*, and *daf-16* were obtained from ref. ^61^, while clones targeting *atf-7*, *zip-7*, *nhr-2*, *lin-39*, *lin-32*, and *dcr-1* were taken from ref. ^62^. For *lin-39*, *lin-32*, *unc-3*, *unc-4*, *elg-5*, and *cfi-1* we used two RNAi clones, taking one from ref. ^61^ and one from ref. ^62^. The *lin-39* RNAi clone used in most figures was the one taken from ref. ^62^. HT115 with the empty plasmid L4440 was used as the control. RNAi feeding was initiated at the L1 stage, unless noted otherwise. Stage-specific RNAi of *lin-39* was conducted as described in ref. ^54^. Details can be found in the paragraph “Lifespan assays”. In several of the experiments we used *eri-1(mg366)* mutant animals to enhance efficacy of RNAi. Use of this background is always stated in the respective figure panels, figure legends, and/or text.

### Lifespan assays

6-well plates were pre-seeded with 100 µl of 10× concentrated bacteria. *C. elegans* were synchronized at the L1 stage by bleaching and hedging in M9, then ~40 animals were seeded into each well. Four wells were prepared for each condition. Initially, the animals were grown at 15 °C until they reached the late L4 stage. This assured that any animals carrying the *daf-2(e1370)* allele would progress well through development, without a tendency to enter the dauer state. Next, plates were shifted to 20 °C and 5-fluoro-2′-deoxyuridine (FUDR) was added to a final concentration of 50 µM to prevent progeny production. Animals were censored and death incidents were scored every 2–3 days by using a platinum wire, as described also in ref. ^63^.

For lifespan experiments using stage-specific RNAi during development or adulthood, we proceeded as follows: For each condition, ~2000 synchronized L1 animals were seeded onto a total of four 9 cm plates, each of which was pre-seeded with 1 ml of 10× concentrated RNAi bacteria. For RNAi throughout life or for RNAi only during development, animals were grown on the RNAi bacteria of interest at 15 °C, while animals predetermined for RNAi only during adulthood were grown on HT115 bacteria at 15 °C. At the L4 stage, animals were collected and washed with M9 until the supernatant (SN) was clear of bacteria. SN was removed, worms were resuspended in 10 ml of Worm-Wash, containing 5 µg/ml Tetracycline, 50 µg/ml Carbenicillin, and 200 µg/ml Streptomycin, and then rotated for 20 min at room temperature (RT). Animals were pelleted, the Worm-Wash replaced, and the animals rotated for another 20 min at RT. Finally, this washing step was repeated for a third time, but now in 10 ml of M9 containing just 50 µg/ml of carbenicillin. SN was partially removed, and for each condition four wells of a 6-well plate were seeded with 40 worms. For RNAi throughout life or for RNAi only during adulthood, these wells were pre-seeded with 100 µl of the respective 10x concentrated RNAi bacteria. For RNAi only during development, the wells were pre-seeded with 100 µl of 10x concentrated *dcr-1* RNAi bacteria. All these wells also contained 50 µM FUDR. From here on forward, animals were grown at 20 °C for the remainder of the assay. For lifespan experiments using other windows of stage-specific RNAi, i.e. those shown in Fig. 3c, the same protocol was used, but the shifting timepoints to and from the respective bacteria were adjusted accordingly. Timepoints for the addition of FUDR or the shift from 15 °C to 20 °C remained unchanged. During the lifespan assays, researchers were blinded to group allocation.

### Microscopy

*C. elegans* were immobilized using 2,3-butanedione monoxime (BDM), mounted on 2% agarose pads, and imaged with a Zeiss Axio Observer Z1 microscope. Images were captured using either 20× or 40× objectives. At least 10 worms were examined for each condition and representative images were selected for presentation in the respective figures. Some images were rotated or irrelevant image content outside the displayed animal cropped away to improve viewability.

### RNA isolation and mRNA sequencing

6-well plates were pre-seeded with 100 µl of the required 10x concentrated bacteria. *C. elegans* were synchronized at the L1 stage by bleaching and hedging in M9, then ~100 animals were seeded into each well. Four wells were prepared for each condition. Initially, the animals were grown at 15 °C until they reached the late L4 stage. This assured that any animals carrying the *daf-2(e1370)* allele would progress well through development, without a tendency to enter the dauer state. The only difference was made for *glp-4(bn2)* animals. These were grown at 25 °C until the late L4 stage, to prevent germ cell production. For all conditions, animals were then shifted to 20 °C and FUDR was added to a final concentration of 50 µM.

For each condition and timepoint, a minimum of 80 worms were collected in triplicate. Worms were washed twice with M9, resuspended in 100 µl Tri Reagent (Zymo Research, Cat. No.: R2050-1-200), and then immediately frozen in liquid nitrogen. RNA was extracted with the Direct-zol^TM^ RNA MiniPrep kit (Zymo Research, Cat. No.: R2052) according to the manufacturer’s instructions, and RNA amount and quality were determined using a NanoDrop. mRNA-seq libraries were then constructed, using the TruSeq RNA Library Preparation Kit v2 (Illumina, Cat. No.: RS-122-2001/RS-122-2002) according to the manufacturer’s instructions. Libraries were eventually submitted for 50 bp single-end sequencing on an Illumina NextSeq 550.

### ATAC sequencing

*C. elegans* were synchronized at the L1 stage by bleaching and hedging in M9 and ~20,000 animals were seeded onto a total of five 15 cm plates pre-seeded with 2 ml of 50× concentrated RNAi bacteria. Plates were incubated at 15 °C until the animals reached the late L4 stage. FUDR was added to a final concentration of 50 µM to prevent progeny production, and plates were shifted to 25 °C for 12 h to maximally inactivate included *daf-2(e1370)* alleles. ATAC-seq was conducted as described in ref. ^27^ using duplicates for every condition. In brief, day 1 adults were washed off the plates with M9, washed in PBS, and frozen in liquid nitrogen. Nuclei were released by mechanical homogenization^64^, counted by measuring optical density, and then processed as described in ref. ^65^. 10 ng of genomic DNA were used as the input control. Resulting libraries were analyzed by 50 bp pair-end sequencing on an Illumina Next-seq 550.

### Analysis of raw mRNA-seq and ATAC-seq data with Cactus

The analysis of raw ATAC-seq and mRNA-seq data was conducted using Cactus v0.8.4^25^, using default parameters and the following custom parameters: species = ‘worm’, chromatin_state = ‘iHMM.M1K16.worm_L3’, use_input_control = true, diffbind__summits = ‘TRUE’, diffbind__design = ‘FALSE’, diffbind__edger_tagwise = ‘FALSE’, diffbind__min_count = 1, diffbind__background = ‘FALSE’, diffbind__library_size = ‘DBA_LIBSIZE_FULL’, diffbind__scale_control = ‘FALSE’, diffbind__score = ‘DBA_SCORE_TMM_READS_FULL_CPM’, split__peak_assignment = [‘all’, ‘prom’, ‘distNC’, ‘3pUPR’] and pwms_motifs = hughesCePWMSForHomerLO9.motif’. The latter custom motif file contained experimentally defined *C. elegans* TF binding motifs and was created by the Brunet lab and used in ref. ^27^. Two replicates of a genomic DNA control, taken from the strain GR1373, were used in all analyses to remove peaks located in hotspot regions.

### Analysis with R

Downstream analyses were conducted using R (v4.3.0), and the following R packages: ggplot2 (version 3.4.2)^66^, data.table (version 1.14.8)^67^, ggpubr (version 0.6.0)^68^, openxlsx (version 4.2.5)^69^, purrr (version 1.0.1)^70^, magrittr (version 2.0.3)^71^, and GenomicFeatures (version 1.52.0)^72^.

### Preparation of heatmaps

Heatmaps were prepared by using Cactus output files and customizing the Cactus heatmap script to simplify the GO-BPs and chromatin state heatmaps. Heatmap parameters used were: “common__filter_params = c(10, 0, 0, F, 2, ‘ward.D’, T)” and “common__heatmaps_params = c(0.05, F, ‘none’, F, 22, ‘UUDD’, 3.5)”.”common__padj_breaks” was set to “c(0.05, 1e−5, 1e−10, 1e−20, 1e−50)” for Fig. 1 and Supplementary Figs. S1 and S8 and to “c(0.05, 1e−5, 1e−15, 1e−50, 1e−100)” for Supplementary Fig. S3. GO-BP terms were simplified using the *GO_similarity* function from the *simplifyEnrichment* R package (version 1.10.0)^73^ and using the *hdbscan* method^74^. Chromatin states^26^ from the same genomic regions (i.e., “10. PC repressed 1” and “11. PC repressed 2”) were aggregated by taking the sum of the *ov_da* (overlap of Differential Analysis Subsets (DASs^25^) with the chromatin state), *ov_nda* (overlap of background with the chromatin state) and *tot_tgt* (total number of entries in the chromatin state) variables obtained from the Cactus analysis.

### Analysis of the lifespan data

Kaplan-Meier survival estimates were generated using the survfit function from the survival R package^75^. For each condition, 100 bootstrap samples were drawn and a Kaplan–Meier model was fitted. Median survival was interpolated between the two time points nearest to 50% survival. The resulting distribution of bootstrapped medians was used to calculate the median survival and standard error. *P*-values for pairwise comparisons were obtained using two-sided log-rank tests implemented via the survdiff function from the survival R package^75^.

### Processing of ENCODE ChIP-seq data

The LIN-39 CHIP-seq data of L3 stage worms was obtained from the “worm/CHIP” Cactus data folder (Encode experiment ID: ENCSR586WBE). It was annotated in a way similar to what was done in Cactus for DARs, namely by using the *ChIPseeker* function *annotatePeak*^76^, with the arguments: tssRegion = c(−1500, 500), level = ‘gene’, overlap = ‘all’, ignoreOverlap = FALSE, TxDb = txdb. The txdb object was imported from the *txdb.sqlite* file from the Cactus data folder. Distal noncoding peaks were then defined in the same way as in Cactus, where ChIP-seq peak annotations had to be either ‘Distal Intergenic’ or ‘Intron’ and none of the following: ‘Promoter’, ‘5 prime UTR’, ‘3 prime UTR’, or ‘Exon’.

### Processing the neuronal atlas, the tissue-age DEGs, and the *daf-2* mutant single-cell transcriptomic datasets

The scRNA-seq neuronal atlas file, *CeNGEN__Average_integrated_TMM_counts_ lengthNormalized_111521.tsv*, with normalized and integrated gene counts was obtained from refs. ^39,77^. Each neuron was assigned to one or multiple neurotransmitters according to WORMATLAS annotations^43^. For each cell type, highly expressed genes were selected as genes with more than 100 counts. Genes differentially expressed with age in various tissues were obtained from ref. ^37^. Genes up and downregulated with age were selected as genes with a p-value below 0.05 and a fold change higher or lower than 1, respectively. Finally, the gene sets from the neuronal atlas and the age-regulated genes by tissue were overlapped with the genes closest to the ATAC-seq-derived DARs or the CHIP-seq peaks in distal non-coding regions. Significance of the overlap was determined by two-sided Fischer’s exact tests, using the list of all genes evaluated by Cactus as a background. Correction for multiple testing was done by using the p.adjust base R function with the Benjamini and Hochberg’s False Discovery Rate method. We obtained a Seurat object containing the single-cell transcriptomic data of *daf-2* mutants with annotated cell types from the lab of Coleen Murphy. Differential gene expression analysis between *daf-2* mutants and wild-type animals for the VC neurons was performed using the Wilcoxon rank-sum test via Seurat’s FindMarkers function^78^. Genes with adjusted *p*-value < 0.05 were retained for downstream analysis. Gene Ontology enrichment for biological processes was performed using the enrichGO function from the clusterProfiler package^79^, with *C. elegans* annotations from org.Ce.eg.db^80^.

### Reporting summary

Further information on research design is available in the Nature Portfolio Reporting Summary linked to this article.

## Supplementary information

Supplementary Information

Reporting Summary

## Source data

Source data

## Data Availability

The ATAC-seq and mRNA-seq data generated in this study have been deposited in NCBI’s Gene Expression Omnibus under accession numbers GSE271970 and GSE271972. Any *C. elegans* strains used in this study will be available from the Caenorhabditis Genetics Center (CGC) or, if not available from the CGC, from the authors. Source data are provided with this paper.
