# Supplementary material for: LIN-39 is a neuron-specific developmental determinant of longevity in Caenorhabditis elegans with reduced insulin signaling
Source: Nat Commun. 2025 Jul 16;16:6566. doi: 10.1038/s41467-025-61786-y (PMC12267636; doi:10.1038/s41467-025-61786-y)
Supplement: Supplementary file 1 — Supplementary Information [file 41467_2025_61786_MOESM1_ESM.pdf]

Supplementary Information

Supplementary figures

Figure S1

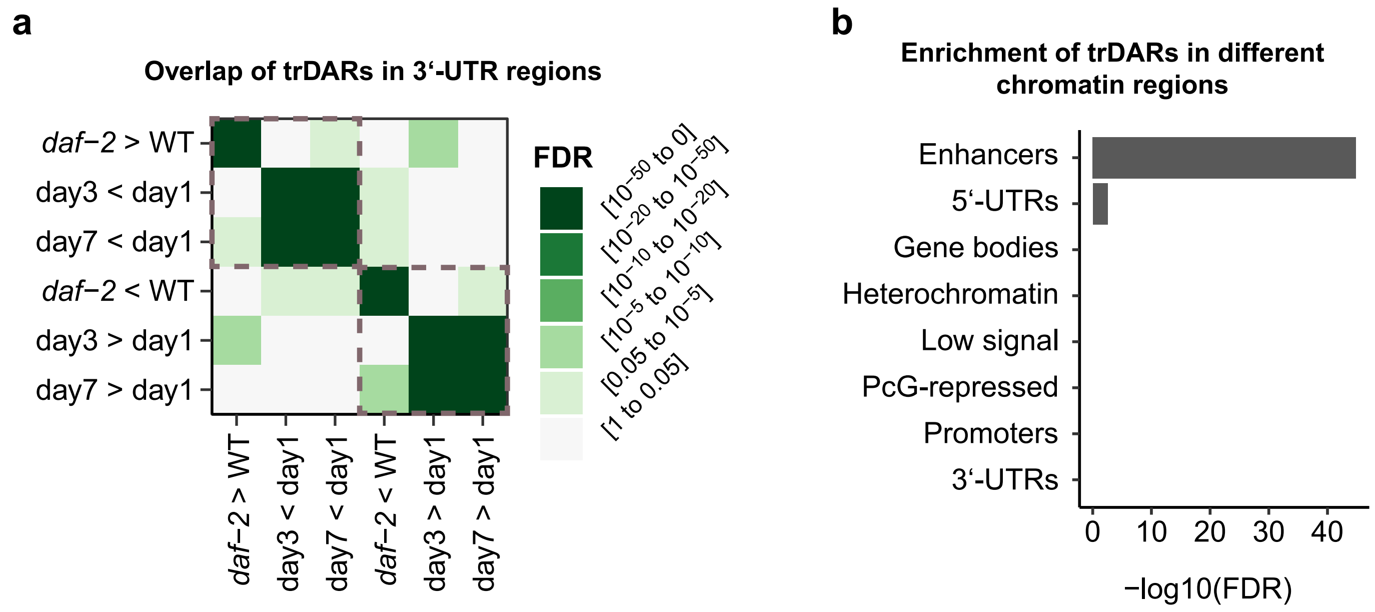
a
Overlap of trDARs in 3‘-UTR regions
daf−2 > WT
day3 < day1
day7 < day1
daf−2 < WT
day3 > day1
day7 > day1
FDR
[10^−50 to 0]
[10^−20 to 10^−50]
[10^−10 to 10^−20]
[10^−5 to 10^−10]
[0.05 to 10^−5]
[1 to 0.05]
b
Enrichment of trDARs in different chromatin regions
Enhancers
5‘-UTRs
Gene bodies
Heterochromatin
Low signal
PcG-repressed
Promoters
3‘-UTRs
0
10
20
30
40
−log10(FDR)

Figure S1

Further characterization of trDARs that arise during aging in wild-type and that arise under reduced insulin/IGF-like signaling (IIS). (a) Heatmap showing the overlap of 3'-UTR-localized trDARs between the indicated conditions. Note: "x > y" refers to regions that are open in x and closed in y, while "x < y" refers to regions that are closed in x and open in y. (b) Barplot showing the enrichment of distNC-localized trDARs that open under reduced IIS in different chromatin regions, adapted from[1]. Abbreviations: trDARs, transcriptionally relevant differentially accessible regions | day1, day3, and day7 correspond to the age of the adult *glp-4* mutants | WT, *eri-1(mg366)* | *daf-2*, *daf-2*(*e1370*); *eri-1(mg366)* | PcG, Polycomb Group | UTR, untranslated regions | FDR, False Discovery Rate. Source data are provided as a Source Data file.

Figure S2

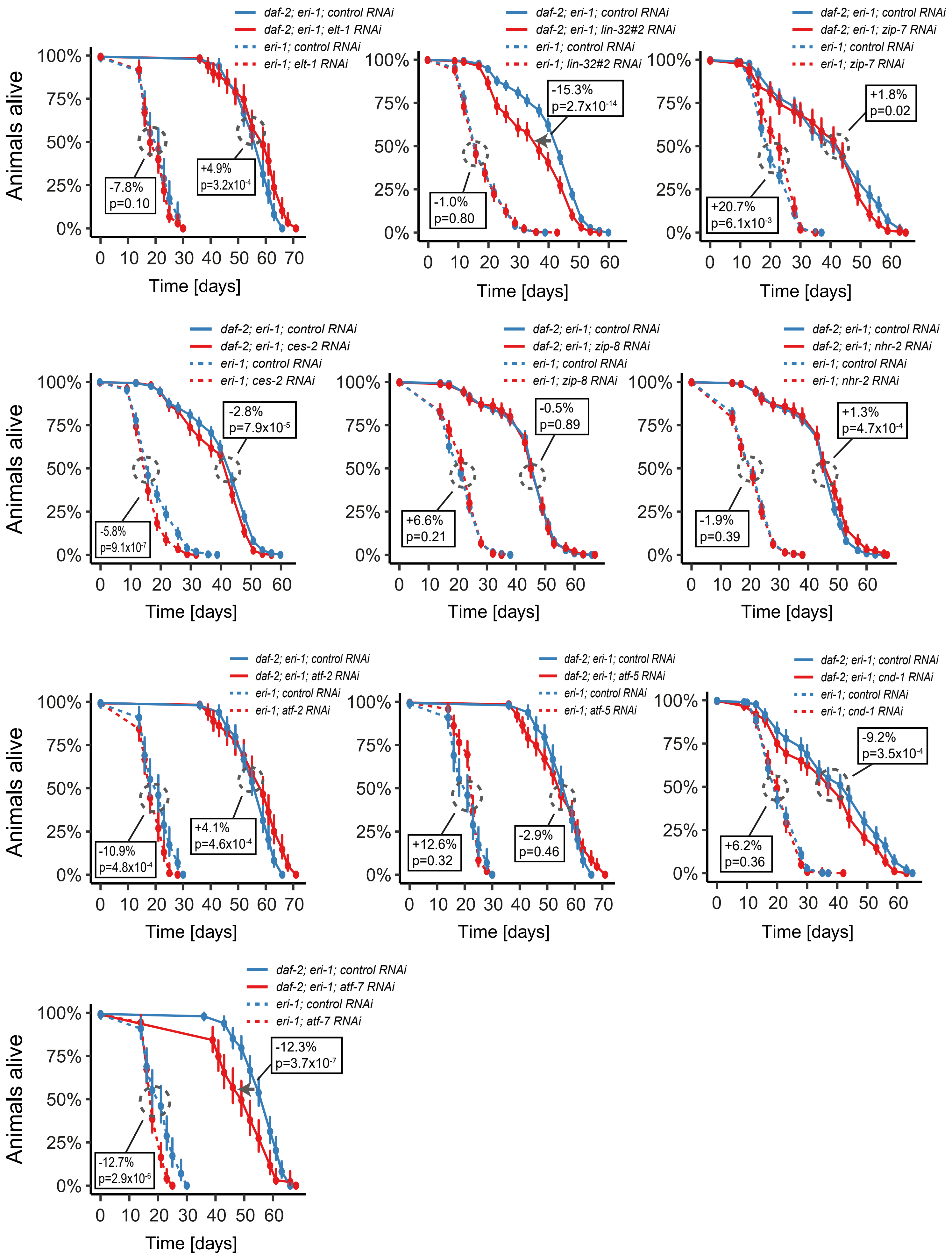

daf-2; eri-1; control RNAi
daf-2; eri-1; elt-1 RNAi
eri-1; control RNAi
eri-1; elt-1 RNAi
Animals alive
100%
75%
50%
25%
0%
-7.8%
p=0.10
+4.9%
p=3.2x10-4
0 10 20 30 40 50 60 70
Time [days]
daf-2; eri-1; control RNAi
daf-2; eri-1; lin-32#2 RNAi
eri-1; control RNAi
eri-1; lin-32#2 RNAi
-15.3%
p=2.7x10-14
-1.0%
p=0.80
0 10 20 30 40 50 60
Time [days]
daf-2; eri-1; control RNAi
daf-2; eri-1; zip-7 RNAi
eri-1; control RNAi
eri-1; zip-7 RNAi
+1.8%
p=0.02
+20.7%
p=6.1x10-3
0 10 20 30 40 50 60
Time [days]
daf-2; eri-1; control RNAi
daf-2; eri-1; ces-2 RNAi
eri-1; control RNAi
eri-1; ces-2 RNAi
-2.8%
p=7.9x10-5
-5.8%
p=9.1x10-7
0 10 20 30 40 50 60
Time [days]
daf-2; eri-1; control RNAi
daf-2; eri-1; zip-8 RNAi
eri-1; control RNAi
eri-1; zip-8 RNAi
-0.5%
p=0.89
+6.6%
p=0.21
0 10 20 30 40 50 60
Time [days]
daf-2; eri-1; control RNAi
daf-2; eri-1; nhr-2 RNAi
eri-1; control RNAi
eri-1; nhr-2 RNAi
+1.3%
p=4.7x10-4
-1.9%
p=0.39
0 10 20 30 40 50 60
Time [days]
daf-2; eri-1; control RNAi
daf-2; eri-1; atf-2 RNAi
eri-1; control RNAi
eri-1; atf-2 RNAi
-10.9%
p=4.8x10-4
+4.1%
p=4.6x10-4
0 10 20 30 40 50 60 70
Time [days]
daf-2; eri-1; control RNAi
daf-2; eri-1; atf-5 RNAi
eri-1; control RNAi
eri-1; atf-5 RNAi
+12.6%
p=0.32
-2.9%
p=0.46
0 10 20 30 40 50 60 70
Time [days]
daf-2; eri-1; control RNAi
daf-2; eri-1; cnd-1 RNAi
eri-1; control RNAi
eri-1; cnd-1 RNAi
-9.2%
p=3.5x10-4
+6.2%
p=0.36
0 10 20 30 40 50 60
Time [days]
daf-2; eri-1; control RNAi
daf-2; eri-1; atf-7 RNAi
eri-1; control RNAi
eri-1; atf-7 RNAi
-12.3%
p=3.7x10-7
-12.7%
p=2.9x10-6
0 10 20 30 40 50 60
Time [days]

Figure S2

Lifespan phenotypes arising from knockdown of additional candidate transcription factors (TFs). Kaplan-Meier survival estimates with 95% confidence intervals for *eri-1(mg366)* and *daf-2(e1370); eri-1(mg366)* mutant *C. elegans* grown from the L1 stage on the indicated RNAi bacteria are shown. “#2” denotes the specific RNAi clone targeting this gene (see also Table S10). (for n, see table S10.) Boxes state changes in median lifespan and the corresponding p-values (based on two-sided log-rank tests) for the indicated comparisons. Source data are provided as a Source Data file.

Figure S3

a

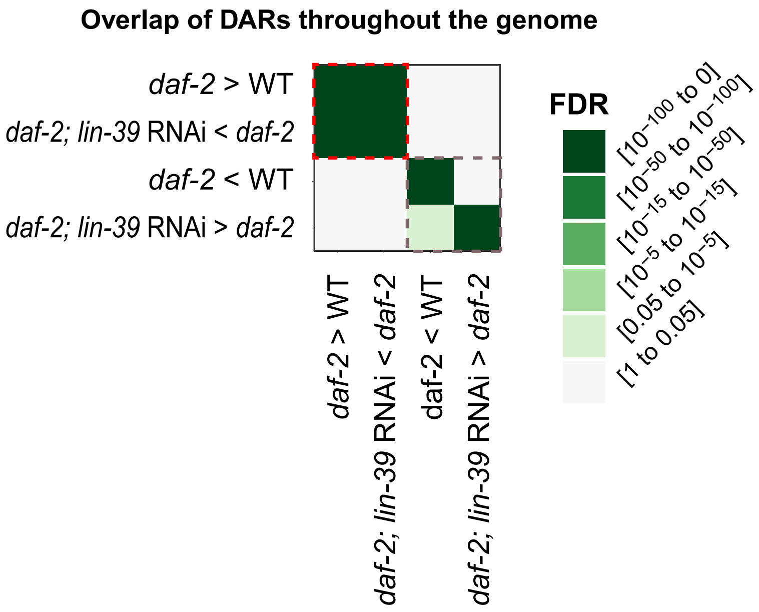

Overlap of DARs throughout the genome
daf-2 > WT
daf-2; lin-39 RNAi < daf-2
daf-2 < WT
daf-2; lin-39 RNAi > daf-2
FDR
[10−100 to 0]
[10−50 to 10−100]
[10−15 to 10−50]
[10−5 to 10−15]
[0.05 to 10−5]
[1 to 0.05]

b

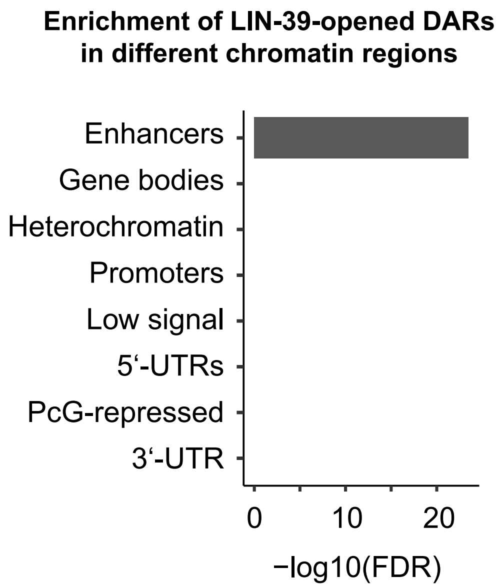

Enrichment of LIN-39-opened DARs in different chromatin regions
Enhancers
Gene bodies
Heterochromatin
Promoters
Low signal
5'-UTRs
PcG-repressed
3'-UTR
0
10
20
−log10(FDR)

Figure S3

LIN-39 is required for the opening of a substantial portion of DARs under reduced IIS. (a) Heatmap showing the overlap of DARs throughout the genome between the indicated conditions. Note: “x > y” refers to regions that are open in x and closed in y, while “x < y” refers to regions that are closed in x and open in y. (b) Barplot showing the enrichment of DARs opened by LIN-39 under reduced IIS in different chromatin regions, adapted from[1]. Abbreviations: WT, *eri-1(mg366)* | *daf-2*, *daf-2*(*e1370); eri-1(mg366)* | PcG, Polycomb Group | FDR, False Discovery Rate. Source data are provided as a Source Data file.

Figure S4

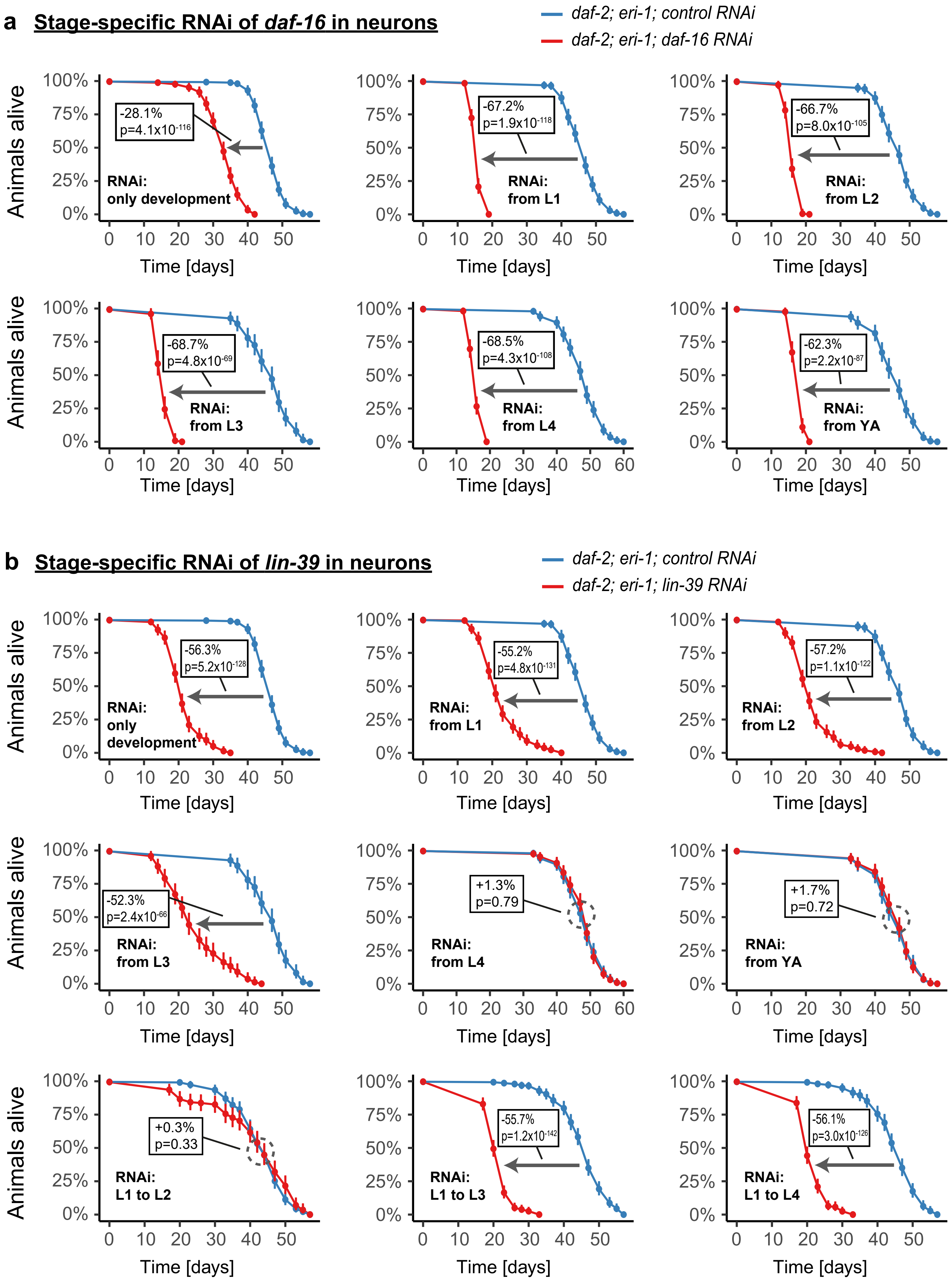

a Stage-specific RNAi of daf-16 in neurons
daf-2; eri-1; control RNAi
daf-2; eri-1; daf-16 RNAi
Animals alive
Time [days]
100%
75%
50%
25%
0%
-28.1% p=4.1x10-116
RNAi: only development
-67.2% p=1.9x10-118
RNAi: from L1
-66.7% p=8.0x10-105
RNAi: from L2
-68.7% p=4.8x10-69
RNAi: from L3
-68.5% p=4.3x10-108
RNAi: from L4
-62.3% p=2.2x10-87
RNAi: from YA
b Stage-specific RNAi of lin-39 in neurons
daf-2; eri-1; control RNAi
daf-2; eri-1; lin-39 RNAi
-56.3% p=5.2x10-128
RNAi: only development
-55.2% p=4.8x10-131
RNAi: from L1
-57.2% p=1.1x10-122
RNAi: from L2
-52.3% p=2.4x10-66
RNAi: from L3
+1.3% p=0.79
RNAi: from L4
+1.7% p=0.72
RNAi: from YA
+0.3% p=0.33
RNAi: L1 to L2
-55.7% p=1.2x10-142
RNAi: L1 to L3
-56.1% p=3.0x10-126
RNAi: L1 to L4

Figure S4

Neuronal LIN-39 acts specifically around the L3 stage, while neuronal DAF-16 acts throughout life to promote longevity under reduced IIS. Kaplan-Meier survival estimates with 95% confidence intervals for *daf-2(e1370); sid-1(pk3321)* mutant *C. elegans* with *sid-1* reconstituted only in the nervous system, resulting in neuron-specific RNAi. Animals were grown on either *daf-16* (a) or *lin-39* (b) RNAi bacteria during the indicated periods – either from an indicated stage onwards or up to an indicated stage. (for n, see table S12.) Boxes state changes in median lifespan and the corresponding p-values (based on two-sided log-rank tests) for the indicated comparisons. Source data are provided as a Source Data file.

Figure S5

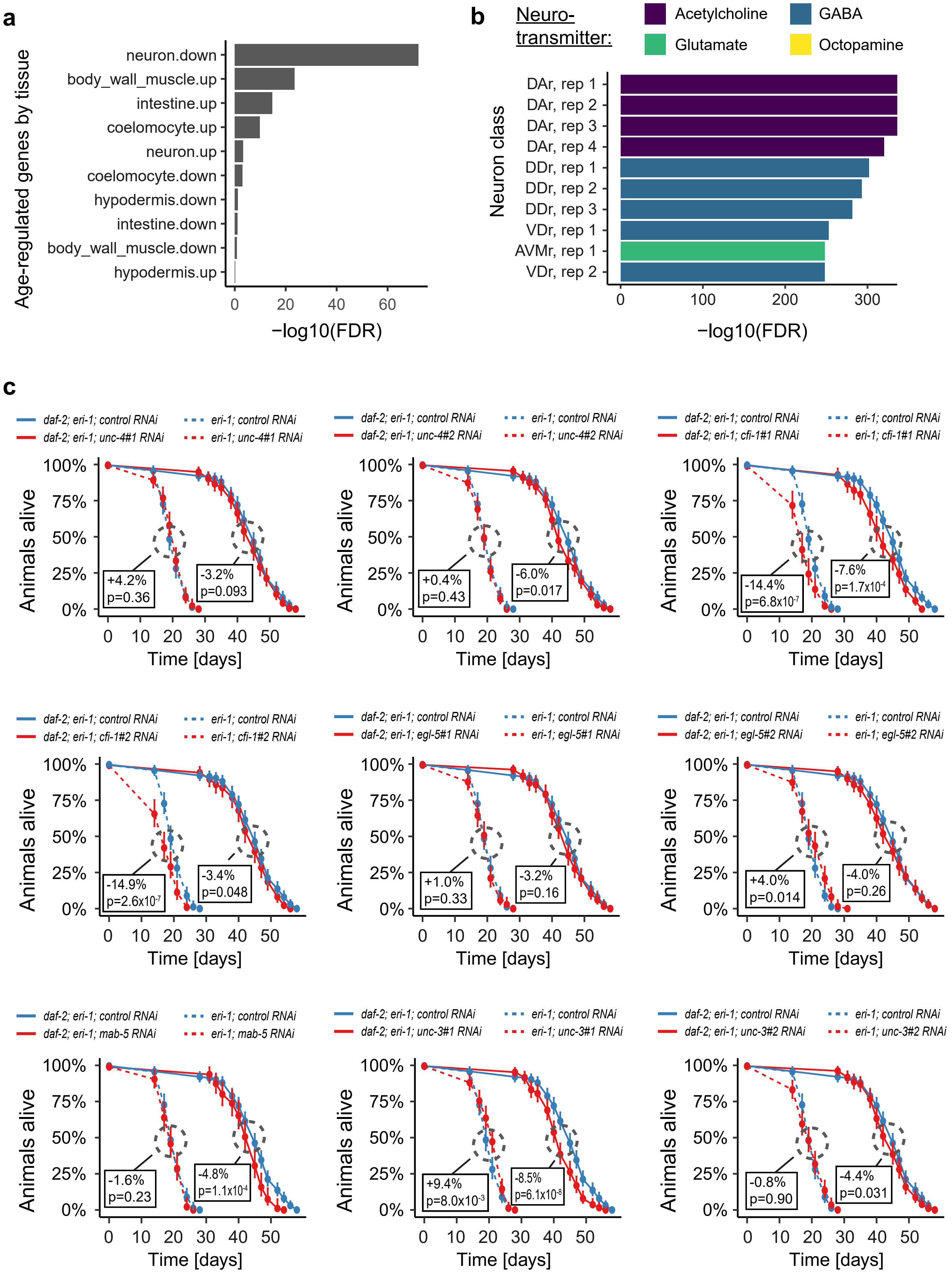
a
Age-regulated genes by tissue
neuron.down
body_wall_muscle.up
intestine.up
coelomocyte.up
neuron.up
coelomocyte.down
hypodermis.down
intestine.down
body_wall_muscle.down
hypodermis.up
0
20
40
60
−log10(FDR)
b
Neuro-transmitter:
Acetylcholine
GABA
Glutamate
Octopamine
Neuron class
DAr, rep 1
DAr, rep 2
DAr, rep 3
DAr, rep 4
DDr, rep 1
DDr, rep 2
DDr, rep 3
VDr, rep 1
AVMr, rep 1
VDr, rep 2
0
100
200
300
−log10(FDR)
c
daf-2; eri-1; control RNAi
eri-1; control RNAi
daf-2; eri-1; unc-4#1 RNAi
eri-1; unc-4#1 RNAi
+4.2%
p=0.36
-3.2%
p=0.093
daf-2; eri-1; unc-4#2 RNAi
eri-1; unc-4#2 RNAi
+0.4%
p=0.43
-6.0%
p=0.017
daf-2; eri-1; cfi-1#1 RNAi
eri-1; cfi-1#1 RNAi
-14.4%
p=6.8x10-7
-7.6%
p=1.7x10-4
daf-2; eri-1; cfi-1#2 RNAi
eri-1; cfi-1#2 RNAi
-14.9%
p=2.6x10-7
-3.4%
p=0.048
daf-2; eri-1; egl-5#1 RNAi
eri-1; egl-5#1 RNAi
+1.0%
p=0.33
-3.2%
p=0.16
daf-2; eri-1; egl-5#2 RNAi
eri-1; egl-5#2 RNAi
+4.0%
p=0.014
-4.0%
p=0.26
daf-2; eri-1; mab-5 RNAi
eri-1; mab-5 RNAi
-1.6%
p=0.23
-4.8%
p=1.1x10-4
daf-2; eri-1; unc-3#1 RNAi
eri-1; unc-3#1 RNAi
+9.4%
p=8.0x10-3
-8.5%
p=6.1x10-8
daf-2; eri-1; unc-3#2 RNAi
eri-1; unc-3#2 RNAi
-0.8%
p=0.90
-4.4%
p=0.031
Animals alive
100%
75%
50%
25%
0%
0
10
20
30
40
50
Time [days]

Figure S5

LIN-39 promotes longevity by modulating expression of genes in cholinergic neurons that are normally down-regulated with age, and the effect of *lin-39* RNAi is not mimicked by knockdown of any other terminal selector of LIN-39 expressing cholinergic neuron classes. (a, b) distNC-localized regions bound by LIN-39 according to[2] were assigned to their closest neighboring gene, resulting in a list of genes predicted to be regulated by LIN-39. (a) A barplot showing the enrichment of these LIN-39-regulated genes in genes differentially regulated with age in various tissues, as reported in[3]. (b) A barplot showing the enrichment of these LIN-39-regulated genes in genes expressed in various *C. elegans* neuron classes, as reported in[4,5]. (c) Kaplan-Meier survival estimates with 95% confidence intervals for *eri-1(mg366)* and *daf-2(e1370); eri-1(mg366)* mutant *C. elegans* grown from the L1 stage on the indicated RNAi bacteria are shown. “#1” and “#2” denote distinct RNAi clones targeting the same respective gene. (for n, see table S19.) Boxes state changes in median lifespan and the corresponding p-values (based on two-sided log-rank tests) for the indicated comparisons. Source data are provided as a Source Data file.

Figure S6

a

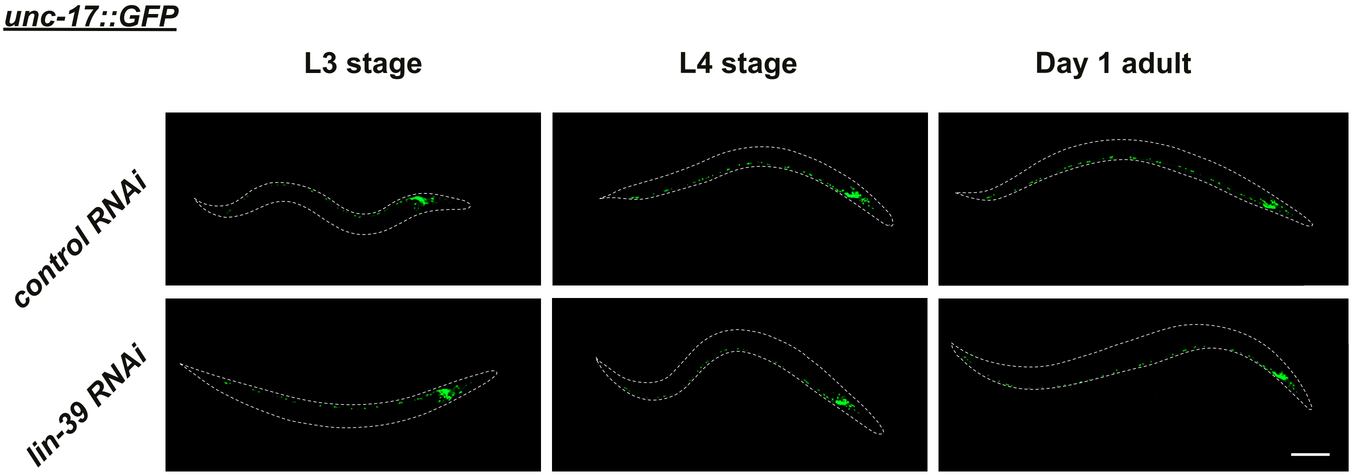

unc-17::GFP
L3 stage
L4 stage
Day 1 adult
control RNAi
lin-39 RNAi

b

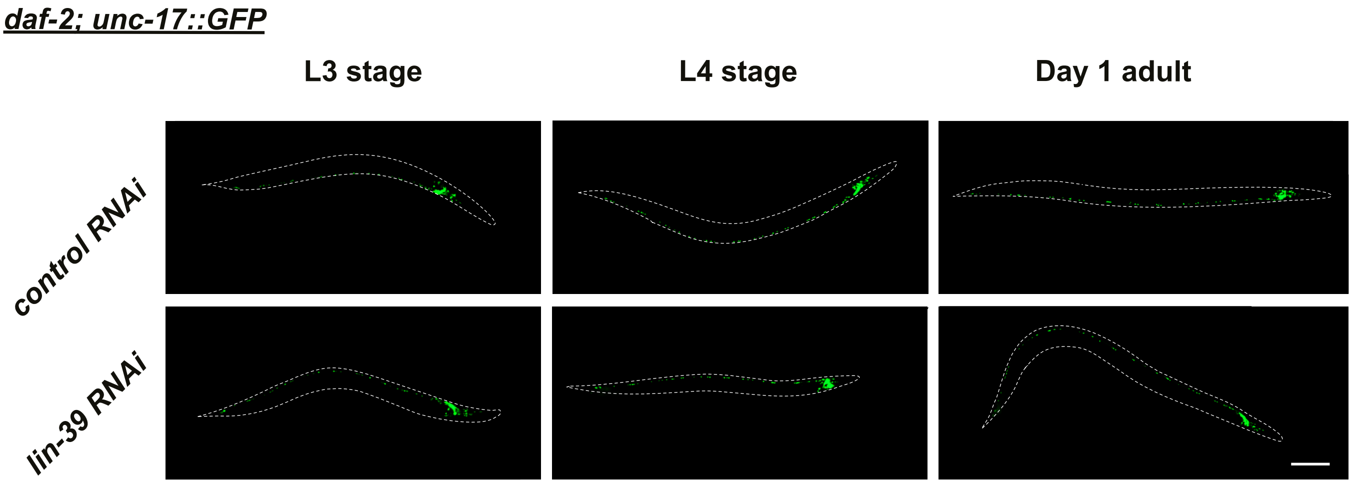

daf-2; unc-17::GFP
L3 stage
L4 stage
Day 1 adult
control RNAi
lin-39 RNAi

Figure S6

Reduced IIS and/or *lin-39* RNAi do not result in substantial changes in the cholinergic nervous system. (a, b) *Punc-17::unc-17::GFP* (a) and *daf-2*; *Punc-17::unc-17::GFP* (b) transgenic *C. elegans* were grown from the L1 stage on the indicated RNAi bacteria and the GFP signal imaged at the indicated stages. White dashed lines indicate the outlines of the animals. (Scale bar: 100 μm)

Figure S7

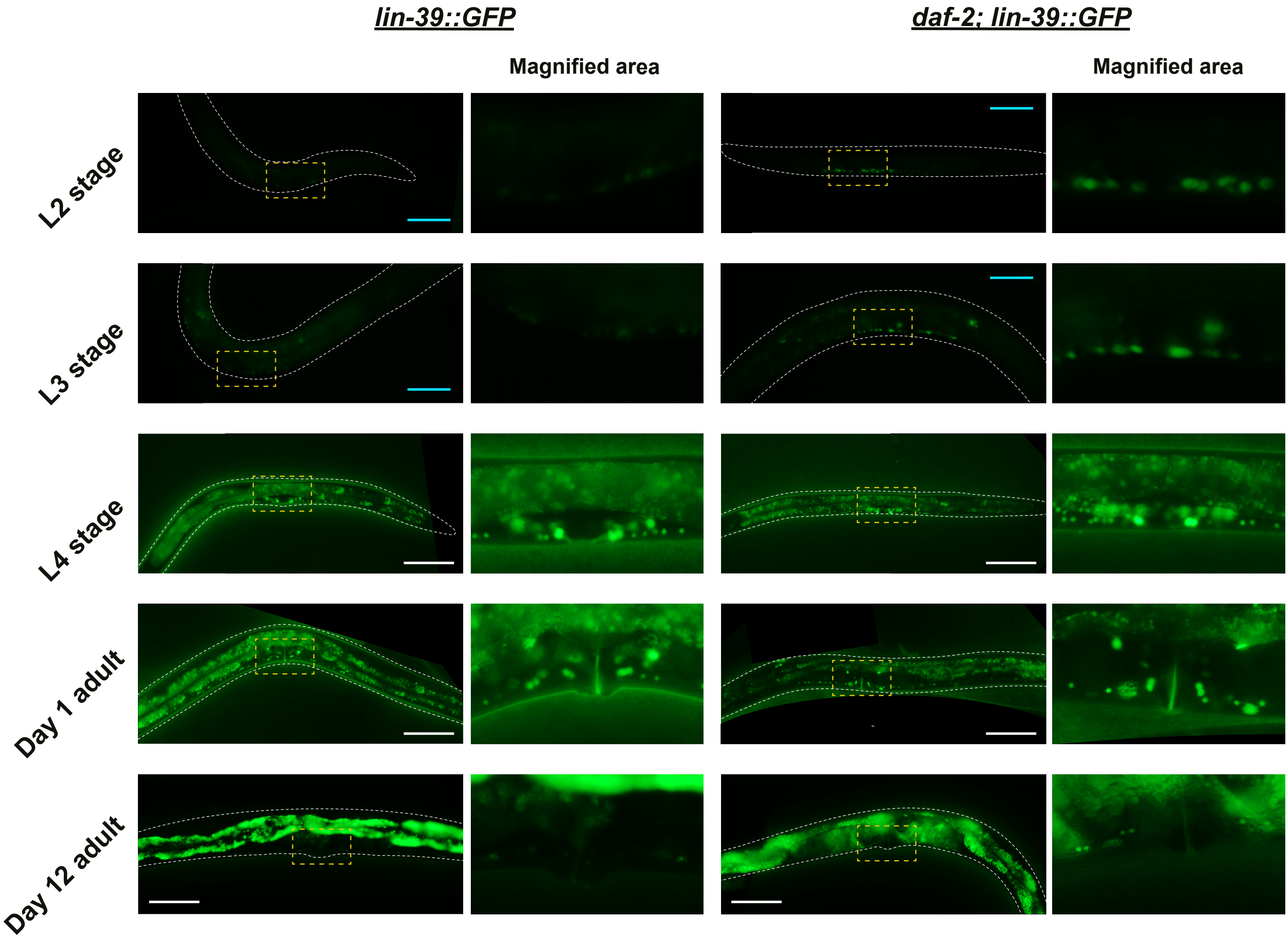

lin-39::GFP
daf-2; lin-39::GFP
Magnified area
Magnified area
L2 stage
L3 stage
L4 stage
Day 1 adult
Day 12 adult

Figure S7

Reduced IIS does not result in substantial changes in the neuronal expression of LIN-39. *Plin-39::lin-39::GFP* and *daf-2*; *Plin-39::lin-39::GFP* transgenic *C. elegans* were grown from the L1 stage on OP-50 bacteria and the GFP signal was imaged at the indicated stages. White dashed lines indicate the outlines of the animals. Yellow dashed frames indicate image areas that are then shown at higher magnification to the right of each respective image. (Blue scale bars: 30 μm; white scale bars: 100 μm)

Figure S8

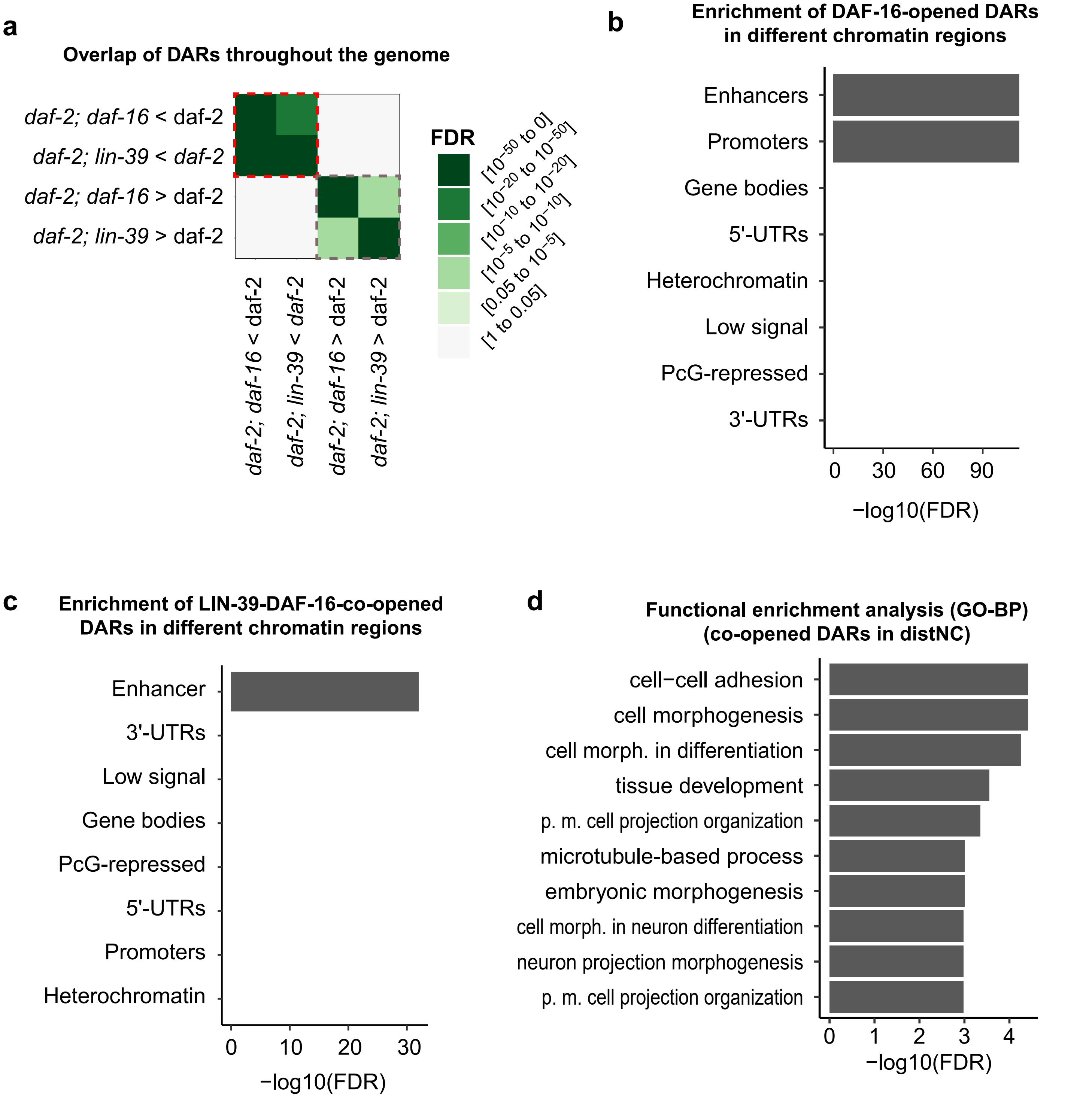
a
Overlap of DARs throughout the genome
daf-2; daf-16 < daf-2
daf-2; lin-39 < daf-2
daf-2; daf-16 > daf-2
daf-2; lin-39 > daf-2
FDR
[10^−50 to 0]
[10^−20 to 10^−50]
[10^−10 to 10^−20]
[10^−5 to 10^−10]
[0.05 to 10^−5]
[1 to 0.05]
b
Enrichment of DAF-16-opened DARs
in different chromatin regions
Enhancers
Promoters
Gene bodies
5'-UTRs
Heterochromatin
Low signal
PcG-repressed
3'-UTRs
0
30
60
90
−log10(FDR)
c
Enrichment of LIN-39-DAF-16-co-opened
DARs in different chromatin regions
Enhancer
3'-UTRs
Low signal
Gene bodies
PcG-repressed
5'-UTRs
Promoters
Heterochromatin
0
10
20
30
−log10(FDR)
d
Functional enrichment analysis (GO-BP)
(co-opened DARs in distNC)
cell−cell adhesion
cell morphogenesis
cell morph. in differentiation
tissue development
p. m. cell projection organization
microtubule-based process
embryonic morphogenesis
cell morph. in neuron differentiation
neuron projection morphogenesis
p. m. cell projection organization
0
1
2
3
4
−log10(FDR)

Figure S8

LIN-39 and DAF-16 promote chromatin opening at the same set of enhancer-localized DARs, which are proximal to genes enriched for neuronal and developmental functions. (a) Heatmap showing the overlap of DARs throughout the genome between the indicated conditions. Note: "x > y" refers to regions that are open in x and closed in y, while "x < y" refers to regions that are closed in x and open in y. (b) Barplot showing the enrichment of DARs opened by DAF-16 under reduced IIS in different chromatin regions, adapted from[1]. (c) Barplot showing the enrichment of DARs that require both LIN-39 and DAF-16 for their opening under low IIS in different chromatin regions, adapted from[1]. (d) Barplot showing a functional enrichment analysis of the genes proximal to distNC-localized DARs co-opened by LIN-39 and DAF-16 under reduced IIS. Some terms were adapted or shortened for improved readability (see Table S25 for the original terms). Abbreviations: WT, *eri-1(mg366)* | *daf-2*, *daf-2*(*e1370); eri-1(mg366)* | PcG, Polycomb Group | UTR, untranslated regions | FDR, False Discovery Rate | p.m., plasma membrane. Source data are provided as a Source Data file.

Figure S9

a

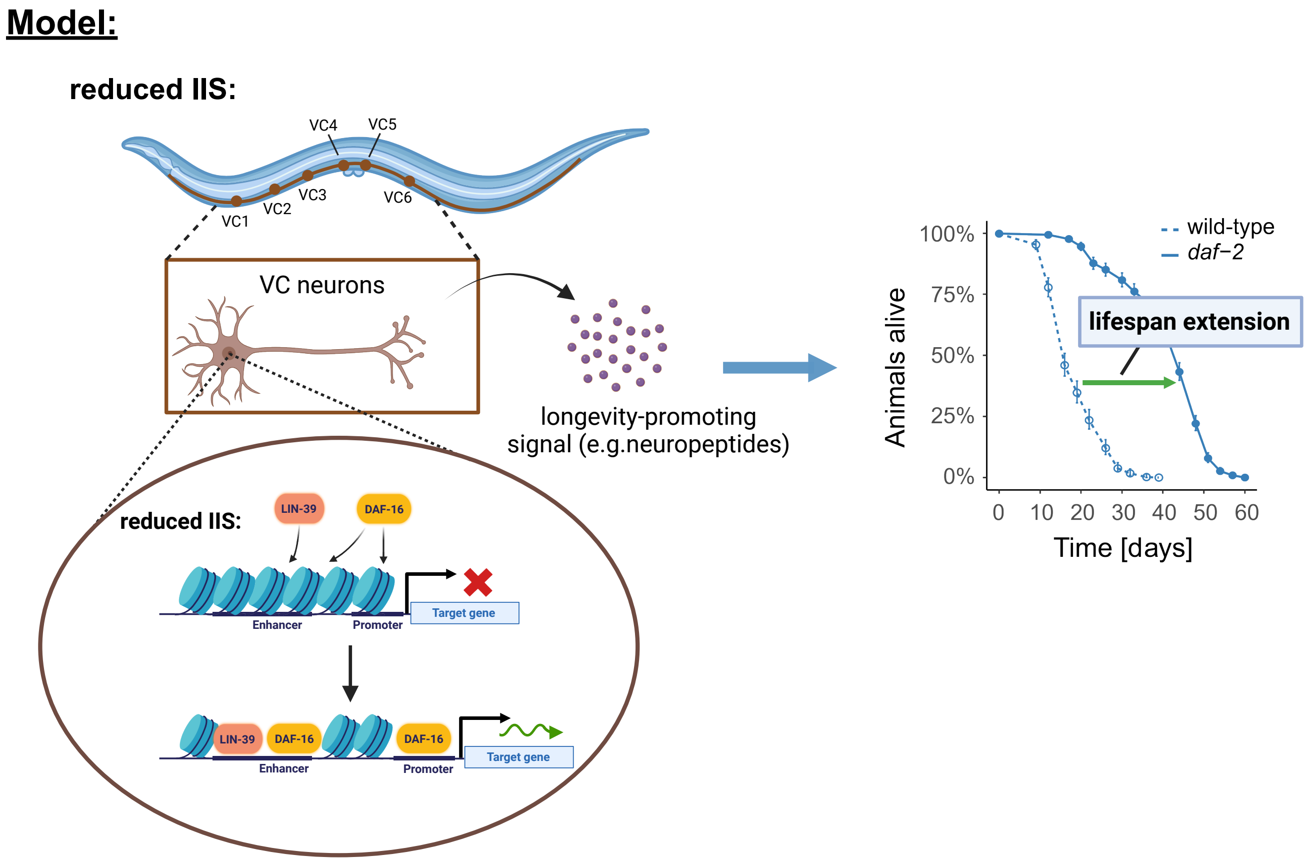

Model:
reduced IIS:
VC4
VC5
VC1
VC2
VC3
VC6
VC neurons
longevity-promoting signal (e.g.neuropeptides)
reduced IIS:
LIN-39
DAF-16
Enhancer
Promoter
Target gene
100%
75%
50%
25%
0%
Animals alive
0 10 20 30 40 50 60
Time [days]
wild-type
daf–2
lifespan extension

b

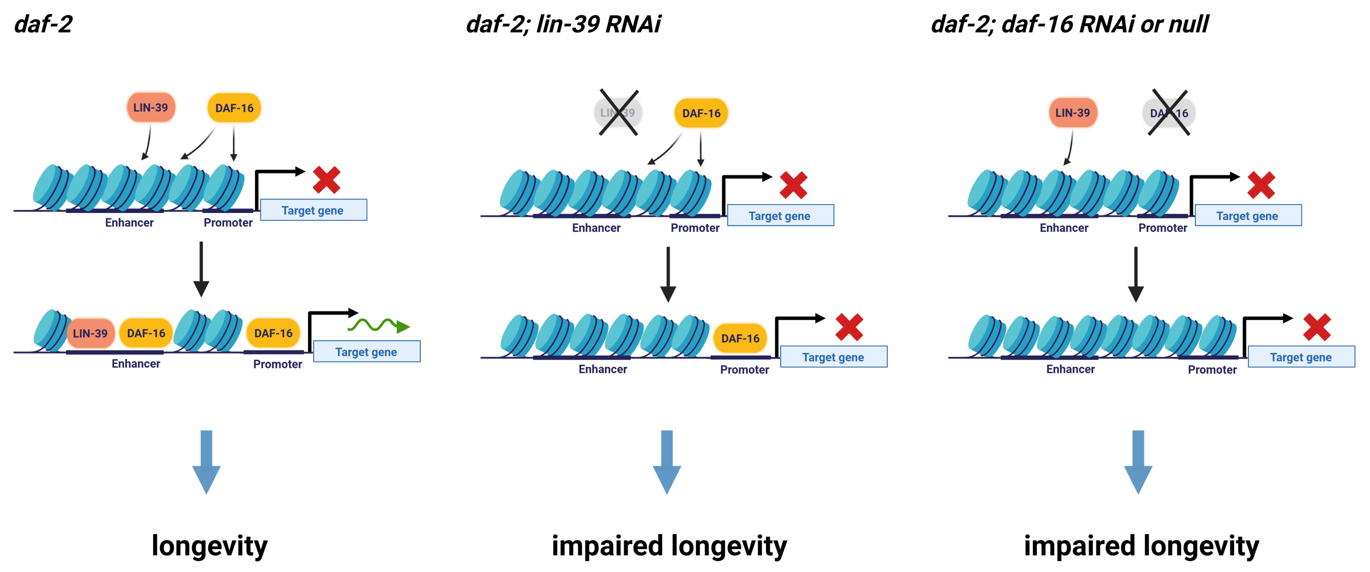

daf-2
daf-2; lin-39 RNAi
daf-2; daf-16 RNAi or null
LIN-39
DAF-16
Enhancer
Promoter
Target gene
longevity
impaired longevity
impaired longevity

Figure S9

Model (a) In *daf-2* animals with reduced IIS, LIN-39 and DAF-16 cooperate in opening enhancers and activating gene expression of proximal genes. These genes determine the characteristics of the cholinergic VC motor neurons. These neurons in turn are required for animals with reduced IIS to become long-lived – presumably due to emission of a longevity-promoting signal in the form of secreted neuropeptides. (b) LIN-39 and DAF-16 need to cooperate to efficiently open and utilize the enhancers mentioned in (a). If any of these TFs is missing, the expression of the nearby genes is impaired, and the neurons fail to acquire their longevity-promoting capabilities. Created in BioRender. Riedel, C. (2025) https://BioRender.com/6fr7m63.

Supplementary tables

Table S1 - Differentially Accessible Regions (DARs) and their overlap, as determined by ATAC-seq

comparison #1*	comparison #2*	n_comparison #1	n_comparison #2	overlap	FDR	significant enrichment/depletion[§]	Log2 Odds Ratio
daf-2 < WT	daf-2 < WT	2850	2850	2850	0.00e+00		
daf-2 < WT	day3 < day1	2850	9968	517	3.86e-01		-0,066589896
daf-2 < WT	day3 > day1	2850	9196	341	1.46e-04	depletion	-0,34073346
daf-2 < WT	day7 < day1	2850	7171	325	4.32e-03	depletion	-0,265948966
daf-2 < WT	day7 > day1	2850	7007	366	3.64e-02	enrichment	0,191808038
daf-2 > WT	daf-2 > WT	3912	3912	3912	0.00e+00		
daf-2 > WT	day3 < day1	3912	9968	1037	2.40e-35	enrichment	0,722923742
daf-2 > WT	day3 > day1	3912	9196	521	3.34e-02	depletion	-0,15708209
daf-2 > WT	day7 < day1	3912	7171	905	1.69e-70	enrichment	1,119113746
daf-2 > WT	day7 > day1	3912	7007	377	1.03e-04	depletion	-0,320398521
day3 < day1	daf-2 < WT	9968	2850	520	4.17e-02	enrichment	0,165952951
day3 < day1	daf-2 > WT	9968	3912	1036	6.57e-30	enrichment	0,703289915
day3 < day1	day3 < day1	9968	9968	9968	0.00e+00		
day3 < day1	day7 < day1	9968	7171	5128	0.00e+00		3,616674488
day3 < day1	day7 > day1	9968	7007	544	0.00e+00		-2,362588021
day3 > day1	daf-2 < WT	9196	2850	355	1.18e-07	depletion	-0,463421816
day3 > day1	daf-2 > WT	9196	3912	525	5.94e-19	depletion	-0,629395824
day3 > day1	day3 > day1	9196	9196	9196	0.00e+00		
day3 > day1	day7 < day1	9196	7171	331	0.00e+00		-3,36031026
day3 > day1	day7 > day1	9196	7007	3470	0.00e+00		2,455874165
day7 < day1	daf-2 < WT	7171	2850	326	1.87e-02	depletion	-0,217336689
day7 < day1	daf-2 > WT	7171	3912	911	1.07e-59	enrichment	1,07874088
day7 < day1	day3 < day1	7171	9968	5131	0.00e+00		3,349435927
day7 < day1	day3 > day1	7171	9196	322	0.00e+00		-2,917515441
day7 < day1	day7 < day1	7171	7171	7171	0.00e+00		
day7 > day1	daf-2 < WT	7007	2850	395	1.61e-02	enrichment	0,213306021
day7 > day1	daf-2 > WT	7007	3912	383	2.36e-19	depletion	-0,716575768
day7 > day1	day3 < day1	7007	9968	541	0.00e+00		-2,959906354
day7 > day1	day3 > day1	7007	9196	3543	0.00e+00		2,891662647
day7 > day1	day7 > day1	7007	7007	7007	0.00e+00		

*: X < Y indicates regions that are closed in X and open in Y. X > Y indicates regions that are open in X and closed in Y.

§: significance cutoff is FDR<0.05.

FDR: False Discovery Rate

WT: wild-type (N2)

Table S2 - Differentially expressed genes and their overlap, as determined by mRNA-seq

comparison #1*	comparison #2*	n_comparison #1	n_comparison #2	overlap	FDR	significant enrichment/depletion§	Log2 Odds Ratio
daf-2 < WT	daf-2 < WT	2960	2960	2960	0.00e+00		
daf-2 < WT	day3 < day1	2960	3399	1424	5.17e-178	enrichment	1,824569755
daf-2 < WT	day3 > day1	2960	3205	857	1.84e-02	enrichment	0,160184328
daf-2 < WT	day7 < day1	2960	3782	1552	1.58e-187	enrichment	1,837345075
daf-2 < WT	day7 > day1	2960	3817	1012	7.84e-04	enrichment	0,218198503
daf-2 > WT	daf-2 > WT	3246	3246	3246	0.00e+00		
daf-2 > WT	day3 < day1	3246	3399	624	1.58e-45	depletion	-0,976791994
daf-2 > WT	day3 > day1	3246	3205	1051	3.61e-15	enrichment	0,507285466
daf-2 > WT	day7 < day1	3246	3782	762	1.25e-31	depletion	-0,769882906
daf-2 > WT	day7 > day1	3246	3817	1115	5.64e-04	enrichment	0,2146238
day3 < day1	daf-2 < WT	3399	2960	1406	5.87e-106	enrichment	1,373793261
day3 < day1	daf-2 > WT	3399	3246	628	1.91e-14	depletion	-0,549037715
day3 < day1	day3 < day1	3399	3399	3399	0.00e+00		
day3 < day1	day7 < day1	3399	3782	2893	0.00e+00		5,345886544
day3 < day1	day7 > day1	3399	3817	377	8.45e-270	depletion	-2,559685117
day3 > day1	daf-2 < WT	3205	2960	846	2.19e-03	depletion	-0,206885101
day3 > day1	daf-2 > WT	3205	3246	1069	1.03e-60	enrichment	1,103019983
day3 > day1	day3 > day1	3205	3205	3205	0.00e+00		
day3 > day1	day7 < day1	3205	3782	412	4.07e-243	depletion	-2,392443568
day3 > day1	day7 > day1	3205	3817	2435	0.00e+00		4,08803616
day7 < day1	daf-2 < WT	3782	2960	1544	3.36e-117	enrichment	1,407799903
day7 < day1	daf-2 > WT	3782	3246	776	8.91e-07	depletion	-0,336069411
day7 < day1	day3 < day1	3782	3399	2907	0.00e+00		5,252630811
day7 < day1	day3 > day1	3782	3205	424	6.92e-214	depletion	-2,233747921
day7 < day1	day7 < day1	3782	3782	3782	0.00e+00		
day7 > day1	daf-2 < WT	3817	2960	1034	4.24e-01		-0,051492922
day7 > day1	daf-2 > WT	3817	3246	1173	3.68e-39	enrichment	0,841523682
day7 > day1	day3 < day1	3817	3399	366	1.31e-316	depletion	-2,749799129
day7 > day1	day3 > day1	3817	3205	2540	0.00e+00		4,179376116
day7 > day1	day7 > day1	3817	3817	3817	0.00e+00		

*: X < Y indicates genes whose expression is upregulated in Y compared to X. X > Y indicates genes whose expression is downregulated in Y compared to X.

§: significance cutoff is FDR<0.05.

FDR: False Discovery Rate

WT: wild-type (N2)

Table S3 - Transcriptionally relevant Differentially Accessible Regions (trDARs) and their overlap, as determined by ATAC-seq and mRNA-seq

comparison #1*	comparison #2*	n_comparison #1	n_comparison #2	overlap	FDR	significant enrichment/depletion[§]	Log2 Odds Ratio
daf-2 < WT	daf-2 < WT	734	734	734	0.00e+00		
daf-2 < WT	day3 < day1	734	3460	69	1.25e-03	enrichment	0,651228021
daf-2 < WT	day3 > day1	734	3258	40	4.15e-01		0,209046574
daf-2 < WT	day7 < day1	734	2839	35	8.64e-01		-0,081101082
daf-2 < WT	day7 > day1	734	2427	46	2.66e-03	enrichment	0,728492395
daf-2 > WT	daf-2 > WT	1052	1052	1052	0.00e+00		
daf-2 > WT	day3 < day1	1052	3460	76	2.25e-01		0,228793349
daf-2 > WT	day3 > day1	1052	3258	83	1.83e-05	enrichment	0,803201557
daf-2 > WT	day7 < day1	1052	2839	80	3.74e-04	enrichment	0,658944691
daf-2 > WT	day7 > day1	1052	2427	52	1.21e-01		0,358967008
day3 < day1	daf-2 < WT	3460	734	69	3.10e-03	enrichment	0,598015331
day3 < day1	daf-2 > WT	3460	1052	76	4.64e-01		0,133346352
day3 < day1	day3 < day1	3460	3460	3460	0.00e+00		
day3 < day1	day7 < day1	3460	2839	1800	0.00e+00		4,972907862
day3 < day1	day7 > day1	3460	2427	1	1.13e-96	depletion	-7,963756976
day3 > day1	daf-2 < WT	3258	734	41	4.82e-01		-0,184808006
day3 > day1	daf-2 > WT	3258	1052	85	1.59e-02	enrichment	0,421792713
day3 > day1	day3 > day1	3258	3258	3258	0.00e+00		
day3 > day1	day7 < day1	3258	2839	12	5.31e-110	depletion	-4,793065454
day3 > day1	day7 > day1	3258	2427	1038	0.00e+00		3,822783289
day7 < day1	daf-2 < WT	2839	734	35	4.43e-01		-0,226531522
day7 < day1	daf-2 > WT	2839	1052	83	1.24e-03	enrichment	0,591937631
day7 < day1	day3 < day1	2839	3460	1804	0.00e+00		4,823692407
day7 < day1	day3 > day1	2839	3258	11	3.66e-84	depletion	-4,555483883
day7 < day1	day7 < day1	2839	2839	2839	0.00e+00		
day7 > day1	daf-2 < WT	2427	734	48	2.32e-02	enrichment	0,542936742
day7 > day1	daf-2 > WT	2427	1052	55	4.28e-01		0,169204916
day7 > day1	day3 < day1	2427	3460	1	9.40e-129	depletion	-8,395856506
day7 > day1	day3 > day1	2427	3258	1068	0.00e+00		4,077168163
day7 > day1	day7 > day1	2427	2427	2427	0.00e+00		

*: X < Y indicates genes whose expression is upregulated in Y compared to X. X > Y indicates genes whose expression is downregulated in Y compared to X.

[§]: significance cutoff is FDR<0.05.

FDR: False Discovery Rate

WT: wild-type (N2)

Table S4 - trDARs and their overlap with different chromatin regions, as determined by ATAC-seq, mRNA-seq, and data from Ho et al.

trDARs*	chromatin regions[#]	n_trDARs	n_genomic regions	overlap	FDR	significant enrichment/depletion[§]	Log2 Odds Ratio
day3 < day1	3'-UTRs	3460	7337	305	3.13e-81	depletion	-1,499798823
day3 > day1	Low signal	3258	6523	1077	5.23e-67	enrichment	1,034079725
day7 < day1	3'-UTRs	2839	7337	282	2.79e-52	depletion	-1,278126871
day3 > day1	Promoters	3258	2661	112	1.57e-47	depletion	-1,721692804
day7 < day1	Gene bodies	2839	965	401	1.09e-41	enrichment	1,239542451
daf-2 > WT	3'-UTRs	1052	7337	115	1.23e-35	depletion	-1,56986358
day7 < day1	PcG-repressed	2839	2033	338	8.63e-28	enrichment	1,064915009
day3 < day1	Enhancers	3460	6496	1338	2.96e-24	enrichment	0,551574177
daf-2 > WT	Enhancers	1052	6496	426	5.57e-24	enrichment	0,957473973
day3 > day1	Enhancers	3258	6496	767	5.31e-23	depletion	-0,598072238
daf-2 < WT	3'-UTRs	734	7337	313	3.48e-22	enrichment	1,084304946
day3 < day1	Gene bodies	3460	965	397	4.96e-20	enrichment	0,80704456
day3 < day1	Promoters	3460	2661	499	5.04e-20	enrichment	0,722205135
day7 > day1	3'-UTRs	2427	7337	663	1.89e-19	enrichment	0,642903257
day3 < day1	Heterochromatin	3460	2654	96	1.25e-18	depletion	-1,20511199
daf-2 < WT	Heterochromatin	734	2654	23	8.49e-13	depletion	-1,813326248
day7 > day1	Low signal	2427	6523	602	1.35e-10	enrichment	0,467783574
daf-2 < WT	Promoters	734	2661	124	6.50e-10	enrichment	0,951530727
day3 > day1	Gene bodies	3258	965	161	4.36e-09	depletion	-0,671260825
daf-2 > WT	5'-UTRs	1052	1667	127	6.07e-09	enrichment	0,867019769
day7 > day1	Promoters	2427	2661	177	6.18e-09	depletion	-0,641038392
day7 > day1	Gene bodies	2427	965	113	9.90e-08	depletion	-0,720740079
day3 < day1	PcG-repressed	3460	2033	314	7.53e-07	enrichment	0,468937732
daf-2 > WT	Promoters	1052	2661	60	3.03e-06	depletion	-0,839212214
day7 > day1	Enhancers	2427	6496	705	1.54e-05	depletion	-0,288433008
day7 > day1	PcG-repressed	2427	2033	113	2.47e-05	depletion	-0,578699334
day7 > day1	5'-UTRs	2427	1667	174	4.25e-05	depletion	-0,465449159
daf-2 < WT	Low signal	734	6523	98	2.53e-04	depletion	-0,560548703
daf-2 < WT	Enhancers	734	6496	153	5.19e-04	depletion	-0,449199431
day3 > day1	Heterochromatin	3258	2654	234	5.97e-04	enrichment	0,366594766
day3 > day1	3'-UTRs	3258	7337	727	1.39e-03	enrichment	0,207664007
daf-2 < WT	Gene bodies	734	965	19	2.98e-03	depletion	-0,947391852
day7 < day1	Heterochromatin	2839	2654	118	8.70e-03	depletion	-0,365725299
daf-2 < WT	PcG-repressed	734	2033	48	1.16e-02	depletion	-0,533653481
day7 > day1	Heterochromatin	2427	2654	149	3.17e-02	enrichment	0,282133455
day7 < day1	Promoters	2839	2661	272	5.52e-02		-0,183849959
day7 < day1	5'-UTRs	2839	1667	296	5.89e-02		0,178550121
day3 < day1	Low signal	3460	6523	678	6.96e-02		-0,119365487
daf-2 > WT	PcG-repressed	1052	2033	112	9.25e-02		0,251533533
day7 < day1	Low signal	2839	6523	591	1.25e-01		0,109309995
daf-2 > WT	Low signal	1052	6523	176	1.58e-01		-0,173075663
daf-2 > WT	Heterochromatin	1052	2654	92	1.61e-01		-0,231301773
daf-2 > WT	Gene bodies	1052	965	57	3.44e-01		0,180161746
day3 > day1	5'-UTRs	3258	1667	299	3.49e-01		-0,088697789
day3 < day1	5'-UTRs	3460	1667	320	4.12e-01		-0,076057217
day7 < day1	Enhancers	2839	6496	917	4.26e-01		-0,049544107
day3 > day1	PcG-repressed	3258	2033	221	6.64e-01		-0,04872072
daf-2 < WT	5'-UTRs	734	1667	51	9.42e-01		-0,041566651

*: X < Y indicates trDARs which are open in Y and closed in X. X > Y indicates trDARs which are closed in Y and open in X.

[#]: Chromatin regions were taken from Ho et al., Nature 2014. Naming of some chromatin regions was slightly adapted to improve readability.

[§]: significance cutoff is FDR<0.05.

FDR: False Discovery Rate

PcG-repressed: Polycomb Group protein-repressed

WT: wild-type (N2)

Table S5 - trDARs in the distNC and their overlap, as determined by ATAC-seq and mRNA-seq

comparison #1*	comparison #2*	n_comparison #1	n_comparison #2	overlap	FDR	significant enrichment/depletion[§]	Log2 Odds Ratio
daf-2 < WT	daf-2 < WT	110	110	110	0.00e+00		
daf-2 < WT	day3 < day1	110	1167	8	5.54e-03	enrichment	2,016581288
daf-2 < WT	day3 > day1	110	672	1	1.00e+00		0,003771509
daf-2 < WT	day7 < day1	110	976	8	2.68e-03	enrichment	2,26997087
daf-2 < WT	day7 > day1	110	503	3	1.19e-01		1,984879296
daf-2 > WT	daf-2 > WT	366	366	366	0.00e+00		
daf-2 > WT	day3 < day1	366	1167	44	3.89e-21	enrichment	2,879657814
daf-2 > WT	day3 > day1	366	672	16	1.06e-06	enrichment	2,372841112
daf-2 > WT	day7 < day1	366	976	45	5.82e-25	enrichment	3,185630746
daf-2 > WT	day7 > day1	366	503	11	1.73e-04	enrichment	2,161786435
day3 < day1	daf-2 < WT	1167	110	8	2.10e-03	enrichment	1,984695459
day3 < day1	daf-2 > WT	1167	366	44	1.69e-18	enrichment	2,617850717
day3 < day1	day3 < day1	1167	1167	1167	0.00e+00		
day3 < day1	day7 < day1	1167	976	628	0.00e+00		6,781488003
day3 < day1	day7 > day1	1167	503	0	6.17e-07		
day3 > day1	daf-2 < WT	672	110	1	1.00e+00		-0,375342032
day3 > day1	daf-2 > WT	672	366	16	8.40e-05	enrichment	1,775234535
day3 > day1	day3 > day1	672	672	672	0.00e+00		
day3 > day1	day7 < day1	672	976	3	1.44e-05	depletion	-2,766839333
day3 > day1	day7 > day1	672	503	219	1.24e-262	enrichment	6,249213327
day7 < day1	daf-2 < WT	976	110	8	9.18e-04	enrichment	2,204054554
day7 < day1	daf-2 > WT	976	366	45	1.46e-21	enrichment	2,880380799
day7 < day1	day3 < day1	976	1167	628	0.00e+00		6,652375467
day7 < day1	day3 > day1	976	672	3	2.14e-04	depletion	-2,451397593
day7 < day1	day7 < day1	976	976	976	0.00e+00		
day7 > day1	daf-2 < WT	503	110	3	9.55e-02		1,634902167
day7 > day1	daf-2 > WT	503	366	11	3.29e-03	enrichment	1,555944657
day7 > day1	day3 < day1	503	1167	0	1.51e-08		
day7 > day1	day3 > day1	503	672	221	2.32e-274	enrichment	6,438977724
day7 > day1	day7 > day1	503	503	503	0.00e+00		

*: X < Y indicates genes whose expression is upregulated in Y compared to X. X > Y indicates genes whose expression is downregulated in Y compared to X.

[§]: significance cutoff is FDR<0.05.

FDR: False Discovery Rate

WT: wild-type (N2)

Table S6 - trDARs in the 3'UTRs and their overlap, as determined by ATAC-seq and mRNA-seq

comparison #1*	comparison #2*	n_comparison #1	n_comparison #2	overlap	FDR	significant enrichment/depletion[§]	Log2 Odds Ratio
daf-2 < WT	daf-2 < WT	95	95	95	5.37e-288		
daf-2 < WT	day3 < day1	95	231	4	3.76e-03	enrichment	3,227712839
daf-2 < WT	day3 > day1	95	279	1	6.04e-01		1,068339354
daf-2 < WT	day7 < day1	95	208	4	2.99e-03	enrichment	3,366626443
daf-2 < WT	day7 > day1	95	193	6	5.17e-06	enrichment	4,362698445
daf-2 > WT	daf-2 > WT	57	57	57	1.36e-184		
daf-2 > WT	day3 < day1	57	231	1	6.84e-01		1,898708289
daf-2 > WT	day3 > day1	57	279	8	4.28e-09	enrichment	5,060269047
daf-2 > WT	day7 < day1	57	208	3	9.24e-03	enrichment	3,688212445
daf-2 > WT	day7 > day1	57	193	2	6.97e-02		3,430419665
day3 < day1	daf-2 < WT	231	95	4	6.60e-03	enrichment	2,809701161
day3 < day1	daf-2 > WT	231	57	1	3.98e-01		1,523211019
day3 < day1	day3 < day1	231	231	231	0.00e+00		
day3 < day1	day7 < day1	231	208	132	5.67e-248	enrichment	9,232627179
day3 < day1	day7 > day1	231	193	0	7.27e-01		
day3 > day1	daf-2 < WT	279	95	1	6.46e-01		0,463178739
day3 > day1	daf-2 > WT	279	57	8	3.21e-08	enrichment	4,50044911
day3 > day1	day3 > day1	279	279	279	0.00e+00		
day3 > day1	day7 < day1	279	208	1	1.00e+00		-0,816283689
day3 > day1	day7 > day1	279	193	81	2.69e-120	enrichment	7,048080021
day7 < day1	daf-2 < WT	208	95	4	5.86e-03	enrichment	2,874741153
day7 < day1	daf-2 > WT	208	57	3	6.59e-03	enrichment	3,436733846
day7 < day1	day3 < day1	208	231	132	1.00e-240	enrichment	9,009982149
day7 < day1	day3 > day1	208	279	1	1.00e+00		-0,808955253
day7 < day1	day7 < day1	208	208	208	0.00e+00		
day7 > day1	daf-2 < WT	193	95	6	5.63e-05	enrichment	3,621576737
day7 > day1	daf-2 > WT	193	57	2	5.40e-02		2,919561276
day7 > day1	day3 < day1	193	231	0	4.57e-01		
day7 > day1	day3 > day1	193	279	83	1.61e-123	enrichment	7,081446398
day7 > day1	day7 > day1	193	193	193	0.00e+00		

*: X < Y indicates genes whose expression is upregulated in Y compared to X. X > Y indicates genes whose expression is downregulated in Y compared to X.

[§]: significance cutoff is FDR<0.05.

FDR: False Discovery Rate

WT: wild-type (N2)

Table S7 - Functional enrichments (GO-BP) of genes closest to trDARs that open in *daf-2* animals

Only the first 30 terms are shown.

GO-BP term	n_genes	FDR	significant enrichment/depletion[§]	Log2 Odds Ratio
nervous system development	36	6.61e-16	enrichment	3,012315379
generation of neurons	33	6.61e-16	enrichment	3,156896941
neurogenesis	33	6.61e-16	enrichment	3,1433087
cell migration	18	4.08e-08	enrichment	2,976776254
localization of cell	18	1.16e-07	enrichment	2,851743391
neuron projection development	19	1.56e-07	enrichment	2,712117686
movement of cell or subcellular component	23	3.07e-07	enrichment	2,352186849
plasma membrane bounded cell projection organization	21	9.13e-07	enrichment	2,35686941
cell adhesion	13	9.13e-07	enrichment	3,168557689
biological adhesion	13	9.13e-07	enrichment	3,168557689
neuron projection morphogenesis	16	1.35e-06	enrichment	2,703330605
plasma membrane bounded cell projection morphogenesis	16	1.35e-06	enrichment	2,703330605
neuron migration	9	1.35e-06	enrichment	3,991383806
cell morphogenesis involved in differentiation	16	1.64e-06	enrichment	2,665105727
cell part morphogenesis	16	1.64e-06	enrichment	2,657576042
cell morphogenesis	17	1.96e-06	enrichment	2,52749315
cell fate commitment	17	1.96e-06	enrichment	2,52749315
post-embryonic development	24	5.38e-06	enrichment	1,979861454
embryonic morphogenesis	13	8.23e-05	enrichment	2,464542027
taxis	14	1.68e-04	enrichment	2,251169113
positive regulation of macromolecule biosynthetic process	16	2.74e-04	enrichment	2,012818265
animal organ development	19	2.75e-04	enrichment	1,818392515
positive regulation of biosynthetic process	16	2.75e-04	enrichment	1,97880983
positive regulation of cellular biosynthetic process	16	2.75e-04	enrichment	1,97880983
positive regulation of transcription, DNA-templated	15	2.75e-04	enrichment	2,057459807
positive regulation of RNA biosynthetic process	15	2.75e-04	enrichment	2,057459807
positive regulation of nucleic acid-templated transcription	15	2.75e-04	enrichment	2,057459807
chemotaxis	13	2.75e-04	enrichment	2,235343676
cell junction assembly	8	3.33e-04	enrichment	3,0087233
positive regulation of RNA metabolic process	15	8.41e-04	enrichment	1,893320611

[§]: significance cutoff is FDR<0.05.
FDR: False Discovery Rate

Table S8 - Enrichment of TF binding motifs in trDARs that open in daf-2 animals

Only the first 30 motifs are shown.

motif*	n_trDARs containing the motif	n_all trDARs	FDR	significant enrichment/depletion§	Log2 Odds Ratio
ces-2	15	366	1.55e-07	enrichment	3,120159802
sani-1	39	366	2.38e-06	enrichment	1,551797284
atf-2	12	366	3.80e-04	enrichment	2,409504078
ces-2	18	366	2.50e-03	enrichment	1,608109894
Y51H4A.4	10	366	2.50e-03	enrichment	2,328293149
F23F12.9	5	366	1.08e-02	enrichment	3,149481267
elt-1	18	366	1.19e-02	enrichment	1,376515124
atf-7	37	366	1.25e-02	enrichment	0,95801875
atf-5	23	366	4.97e-02	enrichment	1,062448881
lin-39	13	366	6.09e-02	enrichment	1,336521773
pax-2	4	366	1.10e-01		2,531405677
W08E12.1	25	366	1.19e-01		0,849790754
die-1	10	366	1.19e-01		-1,146313026
odd-2	1	366	1.30e-01		-2,929168902
ztf-3	39	366	1.86e-01		0,640188103
ceh-22	15	366	1.86e-01		1,057314407
hlh-12	17	366	2.50e-01		0,872091865
hlh-1	8	366	2.72e-01		1,243531892
eor-1	84	366	2.86e-01		0,407876217
let-381	26	366	3.20e-01		0,654997276
pal-1	13	366	3.20e-01		0,92138713
nsy-7	5	366	3.20e-01		1,527273052
blmp-1	73	366	3.40e-01		0,406443762
hsf-1	5	366	3.40e-01		-1,295582165
sex-1	22	366	3.50e-01		0,652745613
mdl-1	7	366	3.50e-01		1,159960832
Y61A9LA.9	7	366	4.43e-01		-1,054642443
tbx-43	6	366	4.44e-01		1,147188723
che-1	3	366	4.65e-01		1,737852627
daf-12	33	366	4.92e-01		0,490883966

*: Motifs were taken from the CISbP database (Weinrauch et al., Cell 2014).

§: significance cutoff is FDR<0.1.

FDR: False Discovery Rate

Table S9 - Enrichment of ChIP-seq peaks in trDARs that open in *daf-2* animals

Only the first 30 chip-seq datasets are shown.

ChIP-seq peaks*	n_trDARs containing a peak	n_all trDARs	FDR	significant enrichment/depletion§	Log2 Odds Ratio
nhr2_MS	101	366	1.55e-55	enrichment	3,390226845
cnd1_MS	66	366	1.58e-21	enrichment	2,38728203
zip8_MS	46	366	1.51e-16	enrichment	2,49582283
unc62_MS	59	366	1.29e-12	enrichment	1,839208126
lin32_MS	36	366	2.75e-11	enrichment	2,268013376
elt1_MS	32	366	3.42e-09	enrichment	2,140695881
hmg4_EE	47	366	2.25e-08	enrichment	1,624266691
lin14_L1	109	366	3.45e-08	enrichment	1,085303374
mls2_MS	42	366	7.87e-08	enrichment	1,64890332
ref2_LE	45	366	4.89e-07	enrichment	1,489280746
ztf11_MS	22	366	6.35e-07	enrichment	2,216196496
sptf1_MS	46	366	7.97e-07	enrichment	1,452308591
ham2_MS	38	366	2.41e-06	enrichment	1,522634214
unc55_LE	44	366	2.49e-06	enrichment	1,407231555
ceh36_EE	32	366	2.94e-06	enrichment	1,652095586
ceh43_MS	25	366	9.46e-06	enrichment	1,804564546
lsl1_YA	5	366	1.12e-05	depletion	-2,49736401
hlh1_MS	48	366	2.17e-05	enrichment	1,224407771
tbx2_LE	51	366	2.45e-05	enrichment	1,171642539
blmp1_L1	80	366	5.32e-05	enrichment	0,918348554
F16B126_L1	2	366	5.75e-04	depletion	-3,061965064
ceh16_L2	23	366	1.03e-03	enrichment	1,428991491
cog1_ME	32	366	1.99e-03	enrichment	1,156395391
efl1_L1_3	2	366	3.06e-03	depletion	-2,85098435
ces1_MS	56	366	3.16e-03	enrichment	0,841572548
ceh34_LE	36	366	3.16e-03	enrichment	1,040512923
snpc34_YA	3	366	3.60e-03	depletion	-2,421503185
daf16_L4_2	32	366	5.07e-03	enrichment	1,052870812
lin15B_L3	9	366	5.41e-03	depletion	-1,53218374
unc130_L4	3	366	6.67e-03	depletion	-2,363863741

*: These are the ChIP-seq peaks for the stated TFs, as published by modEncode (Gerstein et al., Science 2010). The following abbreviations designate the stage of the animals used for the ChIP-seq:

L1: L1 larvae
YA: Young adults
L4: L4 larvae
LE: Late embryos
L3: L3 larvae
L2: L2 larvae
MS: Mixed stage embryos
EE: Early embryos
ME: Midstage embryos
Da: Dauers

§: significance cutoff is FDR<0.05.
FDR: False Discovery Rate

Table S10 - Lifespan analyses of Figures 2 and S2

Figures 2c, 2d, and S2

experiment	strain	RNAi	n_animals	median survival time [days]	% change vs control RNAi	pvalue (logrank test)
exp 1 | Figure 2c, S2	daf-2; eri-1	control	292	41,34954751		
exp 1 | Figure 2c, S2	daf-2; eri-1	cnd-1	271	37,55555556	-9,2	0,000347982
exp 1 | Figure 2c	daf-2; eri-1	lin-32#1	184	38,88235294	-6	0,00616889
exp 1 | Figure 2c	daf-2; eri-1	lin-39#1	269	29,75	-28,1	7,68143E-18
exp 1 | Figure 2c, S2	daf-2; eri-1	zip-7	187	42,1	1,8	0,020419284
exp 1 | Figure 2c, S2	eri-1	control	391	18,74375		
exp 1 | Figure 2c, S2	eri-1	cnd-1	261	19,90425532	6,2	0,363390907
exp 1 | Figure 2c	eri-1	lin-32#1	187	21,23214286	13,3	0,043183885
exp 1 | Figure 2c, S2	eri-1	lin-39#1	202	16,80645161	-10,3	2,10593E-11
exp 1 | Figure 2c, S2	eri-1	zip-7	171	22,625	20,7	0,006068963
exp 2 | Figure 2c, 2d, S2	daf-2; eri-1	control	720	42,59177465		
exp 2 | Figure 2c, S2	daf-2; eri-1	ces-2	367	41,3902439	-2,8	7,90004E-05
exp 2 | Figure 2c, S2	daf-2; eri-1	lin-32#2	378	36,07334526	-15,3	2,68287E-14
exp 2 | Figure 2c, 2d, S2	daf-2; eri-1	lin-39#2	409	30,12162162	-29,3	2,56461E-50
exp 2 | Figure 2c, 2d, S2	eri-1	control	445	15,5		
exp 2 | Figure 2c, S2	eri-1	ces-2	277	14,60868347	-5,8	9,05549E-07
exp 2 | Figure 2c, S2	eri-1	lin-32#2	359	15,34	-1	0,796463233
exp 2 | Figure 2c, 2d, S2	eri-1	lin-39#2	272	16,02240099	3,4	0,068464716
exp 3 | Figure 2c, S2	daf-2; eri-1	control	703	45,28248588		
exp 3 | Figure 2c, S2	daf-2; eri-1	nhr-2	316	45,85994398	1,3	0,00046835
exp 3 | Figure 2c, S2	daf-2; eri-1	zip-8	330	45,05031646	-0,5	0,89197834
exp 3 | Figure 2c, S2	eri-1	control	731	20,23653025		
exp 3 | Figure 2c, S2	eri-1	nhr-2	381	19,84292763	-1,9	0,387547389
exp 3 | Figure 2c, S2	eri-1	zip-8	297	21,58180147	6,6	0,205074592
exp 4 | Figure 2c, S2	daf-2; eri-1	control	146	55,68571429		
exp 4 | Figure 2c, S2	daf-2; eri-1	atf-2	115	57,94117647	4,1	0,000456032
exp 4 | Figure 2c, S2	daf-2; eri-1	atf-5	205	54,04545455	-2,9	0,460156569
exp 4 | Figure 2c, S2	daf-2; eri-1	atf-7	94	48,83281734	-12,3	3,71152E-07
exp 4 | Figure 2c, S2	daf-2; eri-1	elt-1	117	58,4	4,9	0,000315783
exp 4 | Figure 2c, S2	eri-1	control	86	19,71428571		
exp 4 | Figure 2c, S2	eri-1	atf-2	107	17,56	-10,9	0,000482058
exp 4 | Figure 2c, S2	eri-1	atf-5	143	22,20408163	12,6	0,318108371
exp 4 | Figure 2c, S2	eri-1	atf-7	121	17,21621622	-12,7	2,8901E-06
exp 4 | Figure 2c, S2	eri-1	elt-1	132	18,17156863	-7,8	0,104091856

Figure 2e

experiment	strain	n_animals	median survival time [days]	% change vs N2	pvalue (logrank test)
displayed in figure	N2	211	21,52		
displayed in figure	LIN-39 OE	367	24,55	14,1	1,80494E-08
replication	N2	91	15,96875		
replication	LIN-39 OE	105	17	6,5	0,046611615

Table S11 - Lifespan analyses of Figure 3a

experiment	strain	RNAi	n_animals	median survival time [days]	% change vs control RNAi	pvalue (logrank test)
displayed in figure	daf-2 , RNAi in whole body (strain GR1899)	control (L4440)	83	53,14		
displayed in figure	daf-2 , RNAi in whole body (strain GR1899)	lin-39	151	25,17	-52,6	7,84538E-38
displayed in figure	daf-2 , RNAi only in hypodermis (strain IJ415)	control (L4440)	125	40,50		
displayed in figure	daf-2 , RNAi only in hypodermis (strain IJ415)	lin-39	124	38,07	-6	0,044055441
displayed in figure	daf-2 , RNAi only in intestine (strain IJ417)	control (L4440)	291	43,26		
displayed in figure	daf-2 , RNAi only in intestine (strain IJ417)	lin-39	264	42,09	-2,7	0,229926103
displayed in figure	daf-2 , RNAi only in muscles (strain IJ416)	control (L4440)	94	49,71		
displayed in figure	daf-2 , RNAi only in muscles (strain IJ416)	lin-39	69	50,86	2,3	0,483717434
displayed in figure	daf-2 , RNAi only in neurons (strain RIE562)	control (L4440)	174	58,13		
displayed in figure	daf-2 , RNAi only in neurons (strain RIE562)	lin-39	142	20,63	-64,5	2,35685E-82
replication	daf-2 , RNAi in whole body (strain GR1899)	control (L4440)	116	52,71		
replication	daf-2 , RNAi in whole body (strain GR1899)	lin-39	102	25,00	-52,6	6,48436E-33
replication	daf-2 , RNAi only in hypodermis (strain IJ415)	control (L4440)	133	40,02		
replication	daf-2 , RNAi only in hypodermis (strain IJ415)	lin-39	76	33,50	-16,3	0,31199813
replication	daf-2 , RNAi only in intestine (strain IJ417)	control (L4440)	208	39,58		
replication	daf-2 , RNAi only in intestine (strain IJ417)	lin-39	228	40,94	3,5	0,922821835
replication	daf-2 , RNAi only in muscles (strain IJ416)	control (L4440)	75	46,75		
replication	daf-2 , RNAi only in muscles (strain IJ416)	lin-39	61	44,78	-4,2	0,91672242
replication	daf-2 , RNAi only in neurons (strain RIE562)	control (L4440)	124	54,91		
replication	daf-2 , RNAi only in neurons (strain RIE562)	lin-39	89	20,53	-62,6	1,77351E-52

Table S12 - Lifespan analyses of Figures 3b, 3c, and S4

Figure 3b

stage	strain	RNAi	n_animals	median survival time [days]	% change vs control RNAi	pvalue (logrank test)
development and adulthood	daf-2 , RNAi only in neurons (strain RIE562)	lin-39	248	14,75	-67,3	5,3086E-75
development and adulthood	daf-2 , RNAi only in neurons (strain RIE562)	control (L4440)	118	45,11		
Only adult	daf-2 , RNAi only in neurons (strain RIE562)	lin-39	134	40,50	-5,2	0,017725862
Only adult	daf-2 , RNAi only in neurons (strain RIE562)	control (L4440)	121	42,70		
Only development	daf-2 , RNAi only in neurons (strain RIE562)	lin-39	233	17,39	-62,0	1,978E-111
Only development	daf-2 , RNAi only in neurons (strain RIE562)	control (L4440)	215	45,75		

Figures 3c and S4

stage	strain	RNAi	n_animals	median survival time [days]	% change vs control RNAi	pvalue (logrank test)
from L1	daf-2 , RNAi only in neurons (strain RIE562)	control (L4440)	252	45,33		
from L1	daf-2 , RNAi only in neurons (strain RIE562)	daf-16	241	14,87	-67,2	1,905E-118
from L1	daf-2 , RNAi only in neurons (strain RIE562)	lin-39	268	20,32	-55,2	4,8214E-131
from L2	daf-2 , RNAi only in neurons (strain RIE562)	control (L4440)	233	45,96		
from L2	daf-2 , RNAi only in neurons (strain RIE562)	daf-16	204	15,28	-66,7	7,9824E-105
from L2	daf-2 , RNAi only in neurons (strain RIE562)	lin-39	275	19,67	-57,2	1,0981E-122
from L3	daf-2 , RNAi only in neurons (strain RIE562)	control (L4440)	148	46,30		
from L3	daf-2 , RNAi only in neurons (strain RIE562)	daf-16	122	14,51	-68,7	4,78101E-69
from L3	daf-2 , RNAi only in neurons (strain RIE562)	lin-39	166	22,10	-52,3	2,44162E-66
from L4	daf-2 , RNAi only in neurons (strain RIE562)	control (L4440)	235	47,33		
from L4	daf-2 , RNAi only in neurons (strain RIE562)	daf-16	210	14,91	-68,5	4,3282E-108
from L4	daf-2 , RNAi only in neurons (strain RIE562)	lin-39	233	47,96	1,3	0,790634776
from young adulthood (YA)	daf-2 , RNAi only in neurons (strain RIE562)	control (L4440)	193	44,89		
from young adulthood (YA)	daf-2 , RNAi only in neurons (strain RIE562)	daf-16	163	16,93	-62,3	2,18714E-87
from young adulthood (YA)	daf-2 , RNAi only in neurons (strain RIE562)	lin-39	200	45,64	1,7	0,722003505
only development	daf-2 , RNAi only in neurons (strain RIE562)	control (L4440)	254	45,44		
only development	daf-2 , RNAi only in neurons (strain RIE562)	daf-16	251	32,65	-28,1	4,0594E-116
only development	daf-2 , RNAi only in neurons (strain RIE562)	lin-39	219	19,86	-56,3	5,224E-128
L1_to_L2	daf-2 , RNAi only in neurons (strain RIE562)	control (L4440)	230	42,78		
L1_to_L2	daf-2 , RNAi only in neurons (strain RIE562)	lin-39	171	42,89	0,3	0,326275148
L1_to_L3	daf-2 , RNAi only in neurons (strain RIE562)	control (L4440)	291	45,07		
L1_to_L3	daf-2 , RNAi only in neurons (strain RIE562)	lin-39	315	19,96	-55,7	1,1958E-142
L1_to_L4	daf-2 , RNAi only in neurons (strain RIE562)	control (L4440)	256	44,61		
L1_to_L4	daf-2 , RNAi only in neurons (strain RIE562)	lin-39	282	19,57	-56,1	3,0048E-126

Table S13 - DARs affected by lin-39 RNAi and their overlap with DARs affected by reduced IIS

comparison #1*	comparison #2*	n_comparison #1	n_comparison #2	overlap	FDR	significant enrichment/depletion[§]	Log2 Odds Ratio
daf-2(e1370) < WT	daf-2(e1370) < WT	4241	4241	4241	0		
daf-2(e1370) < WT	daf-2(e1370); lin-39(RNAi) < daf-2(e1370)	4241	303	2	1,7641E-15	depletion	-4,515495937
daf-2(e1370) < WT	daf-2(e1370); lin-39(RNAi) > daf-2(e1370)	4241	331	30	0,002849545	enrichment	0,996593282
daf-2(e1370) > WT	daf-2(e1370) > WT	4832	4832	4832	0		
daf-2(e1370) > WT	daf-2(e1370); lin-39(RNAi) < daf-2(e1370)	4832	303	213	1,1638E-106	enrichment	3,882283732
daf-2(e1370) > WT	daf-2(e1370); lin-39(RNAi) > daf-2(e1370)	4832	331	15	0,389553572		-0,443521393
daf-2(e1370); lin-39(RNAi) < daf-2(e1370)	daf-2(e1370) < WT	303	4241	2	3,9026E-10	depletion	-3,986225547
daf-2(e1370); lin-39(RNAi) < daf-2(e1370)	daf-2(e1370) > WT	303	4832	212	5,8709E-109	enrichment	3,899637285
daf-2(e1370); lin-39(RNAi) < daf-2(e1370)	daf-2(e1370); lin-39(RNAi) < daf-2(e1370)	303	303	303	0		
daf-2(e1370); lin-39(RNAi) > daf-2(e1370)	daf-2(e1370) < WT	331	4241	30	1		-0,066936968
daf-2(e1370); lin-39(RNAi) > daf-2(e1370)	daf-2(e1370) > WT	331	4832	13	1,46808E-08	depletion	-2,006183062
daf-2(e1370); lin-39(RNAi) > daf-2(e1370)	daf-2(e1370); lin-39(RNAi) > daf-2(e1370)	331	331	331	0		

*: X < Y indicates DARs which are open in Y and closed in X. X > Y indicates DARs which are closed in Y and open in X.

[§]: significance cutoff is FDR<0.05.

FDR: False Discovery Rate

WT: wild-type (N2)

Table S14 - Overlap of DARs opened by LIN-39 with different chromatin regions, as determined by ATAC-seq and data from Ho et al.

chromatin regions[#]	n_DARs	n_genomic regions	overlap	FDR	significant enrichment/depletion[§]	Log2 Odds Ratio
Enhancers	303	6496	172	3.29e-24	enrichment	1,711828496
3'-UTRs	303	7337	17	5.13e-16	depletion	-2,324000105
PcG-repressed	303	2033	13	3.15e-03	depletion	-1,141493064
Promoters	303	2661	21	3.16e-02	depletion	-0,696535974
Low signal	303	6523	46	1.79e-01		-0,322029595
5'-UTRs	303	1667	29	1.88e-01		0,380640646
Heterochromatin	303	2654	28	9.21e-01		0,006144289
Gene bodies	303	965	15	1.00e+00		-0,08907459

[#]: Chromatin regions were taken from Ho et al., Nature 2014. Naming of some chromatin regions was slightly adapted to improve readability.

[§]: significance cutoff is FDR<0.05.

FDR: False Discovery Rate

PcG-repressed: Polycomb Group protein-repressed

Table S15 - Overlap between genes proximal to DARs openend by LIN-39 and genes regulated in different tissues with age

tissue*	direction of regulation with age*	n_genes at opening DARs	n_genes regulated with age in this set	overlap	p-value	p-values (adjusted)
neuron	down	117	2296	28	9,91539E-05	0,000991539
coelomocyte	down	117	424	0	0,180659023	0,887354447
body_wall_muscle	up	117	410	4	0,305365725	0,887354447
hypodermis	up	117	299	0	0,421272688	0,887354447
hypodermis	down	117	685	2	0,443677224	0,887354447
intestine	up	117	1135	8	0,544419125	0,902070244
intestine	down	117	1566	10	0,726163724	0,902070244
neuron	up	117	1990	12	0,756087695	0,902070244
coelomocyte	up	117	811	5	0,81186322	0,902070244
body_wall_muscle	down	117	845	4	1	1

*: Genes regulated with age in the indicated tissues and the indicated direction according to Wang et al., EMBO Journal 2022
p-values were obtained by two-sided Fischer’s exact test and corrected for multiple testing by the FDR method.

Table S16 - Overlap between genes proximal to LIN-39-bound sites and genes regulated in different tissues with age

tissue*	direction of regulation with age*	n_genes at LIN-39 binding sites[#]	n_genes regulated with age in this set	overlap	p-value	p-values (adjusted)
neuron	down	1526	2296	271	7,15524E-74	7,15524E-73
body_wall_muscle	up	1526	410	53	7,63051E-25	3,81525E-24
intestine	up	1526	1135	92	6,25649E-16	2,0855E-15
coelomocyte	up	1526	811	64	6,45782E-11	1,61446E-10
neuron	up	1526	1990	72	0,00025935	0,0005187
coelomocyte	down	1526	424	34	0,00050214	0,0008369
hypodermis	down	1526	685	46	0,040121115	0,057315879
intestine	down	1526	1566	70	0,06378906	0,079736325
body_wall_muscle	down	1526	845	37	0,120445627	0,133828474
hypodermis	up	1526	299	15	0,73808054	0,73808054

*: Genes regulated with age in the indicated tissues and the indicated direction according to Wang et al., EMBO Journal 2022

[#]: LIN-39 binding sites were derived from modENCODE ChIP-seq data (Gerstein et al., Science 2010)

p-values were obtained by two-sided Fischer’s exact test and corrected for multiple testing by the FDR method.

Table S17 - Overlap between genes proximal to DARs openend by LIN-39 and signature genes of the different neurons

Neuron*	Replicate*	Neurotransmitter*	n_genes at opening DARs	n_genes expressed by neuron*	overlap	p-value	p-values (adjusted)
DA	rep 1	acetylcholine	117	1281	28	5,00436E-10	6,90602E-08
DA	rep 2	acetylcholine	117	1258	25	3,35563E-08	2,22606E-06
DA	rep 3	acetylcholine	117	1304	25	6,26561E-08	2,22606E-06
DD	rep 1	GABA	117	1208	24	6,45234E-08	2,22606E-06
AVM	rep 1	glutamate	117	1329	25	9,27234E-08	2,55916E-06
DD	rep 2	GABA	117	1254	24	1,31948E-07	3,0348E-06
DA	rep 4	acetylcholine	117	1288	23	8,25494E-07	1,6274E-05
AVM	rep 2	glutamate	117	1342	23	1,59537E-06	2,75064E-05
RIC	rep 1	octopamine	117	1353	23	1,7939E-06	2,75064E-05
AVE	rep 1	acetylcholine	117	1376	23	2,37835E-06	2,98374E-05
RIC	rep 2	octopamine	117	1376	23	2,37835E-06	2,98374E-05
DD	rep 3	GABA	117	1194	21	3,42155E-06	3,69873E-05
AVG	rep 1	acetylcholine	117	1513	24	3,48431E-06	3,69873E-05
ASK	rep 1	glutamate	117	1654	25	4,38914E-06	4,32643E-05
AVM	rep 3	glutamate	117	1331	22	5,00108E-06	4,601E-05
VC	rep 1	acetylcholine|serotonine	117	1611	24	1,0813E-05	8,87182E-05
AVG	rep 2	acetylcholine	117	1615	24	1,09291E-05	8,87182E-05
VC	rep 2	acetylcholine|serotonine	117	1432	22	1,62425E-05	0,000124526
RIC	rep 3	octopamine	117	1377	21	2,85855E-05	0,000207621
VC	rep 3	acetylcholine|serotonine	117	1492	22	3,01261E-05	0,00020787
AFD	rep 1	glutamate	117	1544	22	4,78512E-05	0,000314451
VD	rep 1	GABA	117	1110	18	5,5493E-05	0,000348092
AIN	rep 1	acetylcholine	117	1336	20	6,01609E-05	0,000349169
VC	rep 4	acetylcholine|serotonine	117	1562	22	6,0725E-05	0,000349169
RIA	rep 1	glutamate	117	1249	19	7,41069E-05	0,00040907
AIY	rep 1	acetylcholine	117	1375	20	8,16504E-05	0,000417325
RIM	rep 1	glutamate|tyramine	117	1367	20	8,16504E-05	0,000417325
AIN	rep 2	acetylcholine	117	1481	21	8,55357E-05	0,000421569
AVG	rep 3	acetylcholine	117	1703	23	0,000117092	0,000557197
AFD	rep 2	glutamate	117	1550	21	0,000148996	0,00068538
AVA	rep 1	acetylcholine	117	1214	18	0,000154582	0,00068814
ASK	rep 2	glutamate	117	1634	22	0,000173002	0,000746072
VC	rep 5	acetylcholine|serotonine	117	1454	20	0,000185612	0,000776198
RIM	rep 2	glutamate|tyramine	117	1244	18	0,000215637	0,000875232
ASK	rep 3	glutamate	117	1702	22	0,000239813	0,000945547
VD	rep 2	GABA	117	1146	17	0,000255843	0,00098073
IL1	rep 1	glutamate	117	1157	17	0,000281079	0,00104835
VD	rep 3	GABA	117	1162	17	0,000305307	0,001108747
BAG	rep 1	acetylcholine	117	1408	19	0,00034119	0,00114798
SMD	rep 1	acetylcholine	117	1178	17	0,000345057	0,00114798
AVE	rep 2	acetylcholine	117	1291	18	0,000346776	0,00114798
RMD	rep 1	acetylcholine	117	1290	18	0,000356898	0,00114798
IL2	rep 2	acetylcholine	117	1756	22	0,000365638	0,00114798
VC	rep 6	acetylcholine|serotonine	117	1414	19	0,000367074	0,00114798
RMD	rep 2	acetylcholine	117	1294	18	0,000374341	0,00114798
RIA	rep 2	glutamate	117	1207	17	0,000455727	0,00135112
AFD	rep 3	glutamate	117	1529	20	0,00046994	0,00135112
RIA	rep 3	glutamate	117	1214	17	0,000483085	0,00135112
ASER	rep 1	glutamate	117	1669	21	0,0004879	0,00135112
RMD	rep 3	acetylcholine	117	1216	17	0,000506959	0,00135112
PVC	rep 1	acetylcholine	117	892	14	0,000510589	0,00135112
PVD	rep 1	glutamate	117	1562	20	0,000524141	0,00135112
AIY	rep 2	acetylcholine	117	1228	17	0,000526786	0,00135112
SMD	rep 2	acetylcholine	117	1108	16	0,000528699	0,00135112
PVD	rep 2	glutamate	117	1914	23	0,000548524	0,001376297
RIC	rep 4	octopamine	117	1423	19	0,00062246	0,001533918
ASER	rep 2	glutamate	117	1579	20	0,000641629	0,001540348
IL2	rep 3	acetylcholine	117	1707	21	0,000647393	0,001540348
ASER	rep 3	glutamate	117	1584	20	0,000670846	0,001569097
AVE	rep 3	acetylcholine	117	1454	19	0,000718592	0,001652762
ASI	rep 1	NA	117	1464	19	0,000755264	0,001708629
AVA	rep 2	acetylcholine	117	1158	16	0,000790544	0,001759599
ASER	rep 4	glutamate	117	1618	20	0,000817888	0,001791565
SMD	rep 3	acetylcholine	117	1158	16	0,000836904	0,001804575
ASEL	rep 1	glutamate	117	1374	18	0,001011607	0,002136171
AIN	rep 3	acetylcholine	117	1519	19	0,001021647	0,002136171
RIA	rep 4	glutamate	117	1209	16	0,001264715	0,002604935
IL2	rep 4	acetylcholine	117	1775	21	0,001317061	0,002652
ASI	rep 2	NA	117	1426	18	0,001326	0,002652
PVC	rep 2	acetylcholine	117	885	13	0,001452012	0,002862538
RIM	rep 3	glutamate|tyramine	117	1310	17	0,001564689	0,003041227
AWC	rep 1	acetylcholine	117	1313	17	0,001620128	0,003105246
SMD	rep 4	acetylcholine	117	1131	15	0,001823135	0,003446474

RIM	rep 4	glutamate|tyramine	117	1347	17	0,001881107	0,003508011
I5	rep 1	glutamate	117	805	12	0,001922656	0,003537686
VB	rep 1	acetylcholine	117	1211	16	0,002160351	0,003922743
BAG	rep 2	acetylcholine	117	1394	17	0,002387944	0,004279692
AIN	rep 4	acetylcholine	117	1386	17	0,002447647	0,004330453
AWC	rep 2	acetylcholine	117	1251	16	0,00251333	0,004390374
AVK	rep 1	NA	117	1803	20	0,002732868	0,004714197
IL2	rep 5	acetylcholine	117	1657	19	0,003145838	0,005359576
ASI	rep 3	NA	117	1510	18	0,003478873	0,005854689
AWA	rep 1	NA	117	1522	18	0,003566256	0,005929438
AWC	rep 3	acetylcholine	117	1325	16	0,003707906	0,00609156
AIN	rep 5	acetylcholine	117	1336	16	0,004016076	0,006520217
AVA	rep 3	acetylcholine	117	1225	15	0,00455415	0,007307822
ASEL	rep 2	glutamate	117	1376	16	0,004788008	0,007557498
AVK	rep 2	NA	117	1858	20	0,004819274	0,007557498
AVK	rep 3	NA	117	1863	20	0,004911892	0,007616191
AVA	rep 4	acetylcholine	117	1248	15	0,005214613	0,007995739
ASK	rep 4	glutamate	117	1639	18	0,005486613	0,008320358
AWA	rep 2	NA	117	1477	17	0,005625421	0,008438132
AIN	rep 6	acetylcholine	117	1475	17	0,00570127	0,008459949
AVA	rep 5	acetylcholine	117	1267	15	0,005835512	0,008567028
I5	rep 2	glutamate	117	814	11	0,00624131	0,009066324
PHA	rep 1	glutamate	117	821	11	0,006476113	0,009288001
AVH	rep 1	NA	117	1146	14	0,006528522	0,009288001
PVM	rep 1	NA	117	1180	14	0,007678644	0,010812785
AVA	rep 6	acetylcholine	117	1181	14	0,007854453	0,010948632
VB	rep 2	acetylcholine	117	1206	14	0,009242741	0,012754983
BAG	rep 3	acetylcholine	117	1455	16	0,009791502	0,013378488
VB	rep 3	acetylcholine	117	1079	13	0,009892838	0,013384428
ASG	rep 1	glutamate|serotonine	117	1486	16	0,011538451	0,015459283
AVH	rep 2	NA	117	1119	13	0,011987055	0,01577808
AIY	rep 3	acetylcholine	117	1342	15	0,012005061	0,01577808
AWB	rep 1	acetylcholine	117	1148	13	0,014376648	0,018716768
AWA	rep 3	NA	117	1413	15	0,015866661	0,020463544
RMD	rep 4	acetylcholine	117	1241	14	0,016575343	0,021091722
RMD	rep 5	acetylcholine	117	1240	14	0,016659403	0,021091722
AWC	rep 4	acetylcholine	117	1265	14	0,017624061	0,022110185
ADL	rep 1	glutamate	117	1587	16	0,019459062	0,024192347
ASEL	rep 3	glutamate	117	1326	14	0,021134242	0,026040405
AWB	rep 2	acetylcholine	117	1174	13	0,023780101	0,029041185
AVH	rep 3	NA	117	1189	13	0,024748889	0,029959181
PHA	rep 2	glutamate	117	813	10	0,025473997	0,030568796
AWA	rep 4	NA	117	1451	15	0,026540246	0,031573741
ASG	rep 2	glutamate|serotonine	117	1459	15	0,027274531	0,032169959
AWB	rep 3	acetylcholine	117	1240	13	0,029269768	0,034230746
ADL	rep 2	glutamate	117	1563	15	0,031853209	0,036746624
ASG	rep 3	glutamate|serotonine	117	1518	15	0,031953586	0,036746624
ASG	rep 4	glutamate|serotonine	117	1538	15	0,034574043	0,039431553
ASI	rep 4	NA	117	1385	14	0,037701338	0,042645776
AWB	rep 4	acetylcholine	117	1143	12	0,038031321	0,042669287
ADL	rep 3	glutamate	117	1501	14	0,045994991	0,050920727
AWB	rep 5	acetylcholine	117	1198	12	0,046123846	0,050920727
I5	rep 3	glutamate	117	799	9	0,04675226	0,051204856
AVH	rep 4	NA	117	1211	12	0,048475957	0,052674662
OLQ	rep 1	glutamate	117	954	10	0,070549178	0,076060832
NSM	rep 1	serotonine	117	1007	10	0,077004157	0,08237654
ADL	rep 4	glutamate	117	1349	12	0,085033655	0,090266495
OLL	rep 1	glutamate	117	700	7	0,116861278	0,123105774
CAN	rep 1	monoamine	117	836	8	0,147297299	0,153992631
VB	rep 4	acetylcholine	117	1132	10	0,149849412	0,155482849
I5	rep 4	glutamate	117	833	7	0,237277605	0,244360519
NSM	rep 2	serotonine	117	1010	8	0,278455429	0,284643328
RIS	rep 1	GABA	117	1004	8	0,285778887	0,289981517
OLQ	rep 2	glutamate	117	915	7	0,363823174	0,366478817
RIS	rep 2	GABA	117	961	7	0,5041339	0,5041339

*: Genes expressed by the indicated neurons according to Hammarlund et al., Neuron 2018
p-values were obtained by two-sided Fischer’s exact test and corrected for multiple testing by the FDR method.

Table S18 - Overlap between genes proximal to LIN-39-bound sites and signature genes of the different neurons

Neuron*	Replicate*	Neurotransmitter*	n_genes at binding sites	n_genes expressed by neuron*	overlap	p-value	p-values (adjusted)
DA	rep 1	acetylcholine	1526	1281	350	0	0
DA	rep 2	acetylcholine	1526	1288	350	0	0
DA	rep 3	acetylcholine	1526	1258	334	0	0
DA	rep 4	acetylcholine	1526	1304	340	0	0
DD	rep 1	GABA	1526	1254	318	3,9374E-304	1,0867E-302
DD	rep 2	GABA	1526	1208	304	4,1241E-295	9,4853E-294
DD	rep 3	GABA	1526	1194	303	2,2809E-283	4,4966E-282
VD	rep 1	GABA	1526	1162	282	1,0898E-254	1,8799E-253
AVM	rep 1	glutamate	1526	1342	305	6,0395E-250	8,8359E-249
VD	rep 2	GABA	1526	1146	281	6,4029E-250	8,8359E-249
RMD	rep 1	acetylcholine	1526	1290	286	8,7233E-248	1,0944E-246
RMD	rep 2	acetylcholine	1526	1240	278	1,5568E-243	1,7903E-242
AVM	rep 2	glutamate	1526	1329	294	1,7098E-240	1,815E-239
VD	rep 3	GABA	1526	1110	274	2,5068E-238	2,471E-237
AVG	rep 1	acetylcholine	1526	1703	342	8,3459E-234	7,6783E-233
AIN	rep 1	acetylcholine	1526	1475	294	1,9759E-231	1,7042E-230
AVG	rep 2	acetylcholine	1526	1615	329	1,3132E-230	1,066E-229
AIN	rep 2	acetylcholine	1526	1386	286	2,0573E-229	1,5773E-228
AIN	rep 3	acetylcholine	1526	1481	302	2,4512E-229	1,7804E-228
RIM	rep 1	glutamate|tyramine	1526	1367	294	5,9232E-227	4,087E-226
AVM	rep 3	glutamate	1526	1331	290	1,6705E-225	1,0978E-224
AIN	rep 4	acetylcholine	1526	1336	281	1,2961E-224	8,1301E-224
IL2	rep 1	acetylcholine	1526	1775	328	1,7878E-222	1,0727E-221
RMD	rep 3	acetylcholine	1526	1216	268	5,024E-220	2,8888E-219
RIC	rep 1	octopamine	1526	1423	296	1,5554E-219	8,5858E-219
RMD	rep 4	acetylcholine	1526	1241	275	9,9786E-219	5,2964E-218
AVG	rep 3	acetylcholine	1526	1513	312	2,3454E-218	1,1987E-217
PVM	rep 1	NA	1526	1180	261	3,3246E-216	1,6386E-215
VB	rep 1	acetylcholine	1526	1211	274	7,2916E-215	3,4698E-214
RMD	rep 5	acetylcholine	1526	1294	281	2,3052E-214	1,0604E-213
IL1	rep 2	glutamate	1526	1157	251	2,4502E-212	1,0907E-211
IL2	rep 3	acetylcholine	1526	1756	315	1,194E-211	5,1491E-211
RIC	rep 2	octopamine	1526	1353	274	2,9474E-211	1,2325E-210
ASK	rep 1	glutamate	1526	1654	299	2,0823E-209	8,4516E-209
IL2	rep 4	acetylcholine	1526	1707	304	1,0146E-204	4,0004E-204
RIC	rep 3	octopamine	1526	1377	286	1,3915E-201	5,334E-201
RIM	rep 2	glutamate|tyramine	1526	1310	277	4,6955E-201	1,7513E-200
VC	rep 1	acetylcholine|serotonine	1526	1611	297	2,2261E-200	8,0843E-200
BAG	rep 1	acetylcholine	1526	1408	270	4,3335E-200	1,5334E-199
AIN	rep 5	acetylcholine	1526	1519	287	2,9663E-199	1,0234E-198
SMD	rep 1	acetylcholine	1526	1178	257	2,4422E-197	8,2199E-197
AVE	rep 1	acetylcholine	1526	1376	274	3,1235E-197	1,0263E-196
RIM	rep 3	glutamate|tyramine	1526	1347	280	1,0217E-196	3,2789E-196
ASK	rep 2	glutamate	1526	1634	291	3,057E-196	9,588E-196
AVH	rep 1	NA	1526	1211	259	3,8347E-196	1,176E-195
AVE	rep 2	acetylcholine	1526	1291	268	1,3406E-195	4,0217E-195
BAG	rep 2	acetylcholine	1526	1455	271	2,0111E-195	5,905E-195
RIM	rep 4	glutamate|tyramine	1526	1244	265	1,5437E-194	4,438E-194
AVH	rep 2	NA	1526	1189	255	2,2559E-194	6,3532E-194
RIC	rep 4	octopamine	1526	1376	275	1,4507E-193	4,004E-193
SMD	rep 2	acetylcholine	1526	1158	250	6,228E-193	1,6852E-192
VB	rep 2	acetylcholine	1526	1206	258	2,116E-192	5,6157E-192
IL2	rep 5	acetylcholine	1526	1657	290	1,1559E-190	3,0098E-190
AIN	rep 6	acetylcholine	1526	1336	271	8,1819E-189	2,0909E-188
BAG	rep 3	acetylcholine	1526	1394	264	5,4059E-188	1,3564E-187
ASK	rep 3	glutamate	1526	1702	295	1,2264E-187	3,0223E-187
AFD	rep 1	glutamate	1526	1544	288	3,5737E-185	8,6521E-185
SMD	rep 3	acetylcholine	1526	1131	246	8,3132E-185	1,978E-184
PVD	rep 1	glutamate	1526	1914	334	1,2514E-182	2,9271E-182
VC	rep 2	acetylcholine|serotonine	1526	1492	274	1,5496E-182	3,564E-182
ASK	rep 4	glutamate	1526	1639	289	1,479E-178	3,346E-178
AVH	rep 3	NA	1526	1146	242	1,5917E-178	3,5428E-178
ASI	rep 1	NA	1526	1464	276	5,8564E-178	1,2828E-177
ASI	rep 2	NA	1526	1426	266	1,9455E-177	4,1951E-177
AVA	rep 1	acetylcholine	1526	1214	250	2,1695E-176	4,606E-176
AVE	rep 3	acetylcholine	1526	1454	276	2,5137E-176	5,256E-176
PVD	rep 2	glutamate	1526	1562	293	3,2025E-176	6,5963E-176
SMD	rep 4	acetylcholine	1526	1108	238	4,3573E-175	8,8427E-175
AFD	rep 2	glutamate	1526	1529	279	3,2714E-174	6,5427E-174
AFD	rep 3	glutamate	1526	1550	278	8,6205E-174	1,6995E-173
AVH	rep 4	NA	1526	1119	237	1,4234E-173	2,7666E-173
VC	rep 3	acetylcholine|serotonine	1526	1562	276	4,3358E-172	8,3103E-172
VC	rep 4	acetylcholine|serotonine	1526	1454	260	1,2076E-169	2,2829E-169
ASER	rep 1	glutamate	1526	1618	281	1,2134E-165	2,2629E-165
ASI	rep 3	NA	1526	1510	268	3,198E-165	5,8844E-165

VB	rep 3	acetylcholine	1526	1132	236	1,4837E-163	2,694E-163
ASER	rep 2	glutamate	1526	1669	285	3,0626E-160	5,4888E-160
AVA	rep 2	acetylcholine	1526	1267	244	3,6435E-157	6,4462E-157
ASER	rep 3	glutamate	1526	1579	270	3,4423E-156	6,0131E-156
AWB	rep 1	acetylcholine	1526	1240	232	5,4943E-156	9,4777E-156
RIA	rep 1	glutamate	1526	1249	245	8,6258E-156	1,4696E-155
ASI	rep 4	NA	1526	1385	245	1,8447E-154	3,1046E-154
AIY	rep 1	acetylcholine	1526	1375	252	4,31E-154	7,166E-154
ASEL	rep 1	glutamate	1526	1374	252	7,5585E-154	1,2418E-153
AWC	rep 1	acetylcholine	1526	1313	237	9,8821E-154	1,6044E-153
AWB	rep 2	acetylcholine	1526	1174	221	1,963E-153	3,15E-153
AVA	rep 3	acetylcholine	1526	1158	232	9,9665E-153	1,5809E-152
ASER	rep 4	glutamate	1526	1584	270	1,0906E-152	1,7103E-152
AWB	rep 3	acetylcholine	1526	1198	225	5,8379E-152	9,052E-152
AWB	rep 4	acetylcholine	1526	1143	218	2,788E-151	4,2749E-151
ASG	rep 1	glutamate|serotonine	1526	1538	264	1,0821E-149	1,641E-149
VC	rep 5	acetylcholine|serotonine	1526	1432	249	1,3913E-149	2,087E-149
VB	rep 4	acetylcholine	1526	1079	222	4,958E-149	7,357E-149
AVA	rep 4	acetylcholine	1526	1181	234	6,1762E-149	9,0671E-149
PVC	rep 1	acetylcholine	1526	892	191	9,5984E-147	1,3943E-146
RIA	rep 2	glutamate	1526	1214	233	1,5764E-146	2,2661E-146
VC	rep 6	acetylcholine|serotonine	1526	1414	247	3,401E-146	4,8385E-146
RIA	rep 3	glutamate	1526	1209	232	5,1909E-146	7,3096E-146
ASG	rep 2	glutamate|serotonine	1526	1518	257	7,6569E-145	1,0673E-144
AIY	rep 2	acetylcholine	1526	1228	224	1,4928E-143	2,0601E-143
AVA	rep 5	acetylcholine	1526	1225	233	7,8466E-143	1,0721E-142
AVA	rep 6	acetylcholine	1526	1248	235	1,7168E-141	2,3228E-141
AWA	rep 1	NA	1526	1522	251	2,6444E-140	3,543E-140
AWC	rep 2	acetylcholine	1526	1325	229	4,1609E-139	5,5212E-139
AWC	rep 3	acetylcholine	1526	1265	227	1,1641E-138	1,53E-138
AWB	rep 5	acetylcholine	1526	1148	215	2,4792E-138	3,2277E-138
AWA	rep 2	NA	1526	1413	233	1,2304E-137	1,5869E-137
AWA	rep 3	NA	1526	1451	238	3,2534E-137	4,1571E-137
PVC	rep 2	acetylcholine	1526	885	183	3,3826E-137	4,2826E-137
ADL	rep 1	glutamate	1526	1563	244	6,3673E-137	7,988E-137
AWC	rep 4	acetylcholine	1526	1251	220	3,7374E-134	4,6465E-134
ADL	rep 2	glutamate	1526	1587	242	1,2494E-133	1,5395E-133
AIY	rep 3	acetylcholine	1526	1342	237	1,3495E-133	1,6481E-133
AWA	rep 4	NA	1526	1477	240	4,1008E-132	4,9641E-132
ASG	rep 3	glutamate|serotonine	1526	1486	245	9,1324E-130	1,0959E-129
ASEL	rep 2	glutamate	1526	1376	233	1,4238E-129	1,6938E-129
ASG	rep 4	glutamate|serotonine	1526	1459	234	3,3232E-128	3,9197E-128
ASEL	rep 3	glutamate	1526	1326	223	1,2931E-127	1,5122E-127
ADL	rep 3	glutamate	1526	1501	229	1,1013E-124	1,2771E-124
OLL	rep 1	glutamate	1526	700	152	1,9191E-120	2,2069E-120
NSM	rep 1	serotonine	1526	1007	176	1,1616E-117	1,3248E-117
RIA	rep 4	glutamate	1526	1207	219	1,227E-117	1,3879E-117
CAN	rep 1	monoamine	1526	836	163	1,2195E-114	1,3682E-114
ADL	rep 4	glutamate	1526	1349	197	8,62E-105	9,5933E-105
AVK	rep 1	NA	1526	1863	249	2,7732E-102	3,0616E-102
OLQ	rep 1	glutamate	1526	954	175	2,1272E-101	2,3298E-101
AVK	rep 2	NA	1526	1858	247	1,4023E-97	1,52376E-97
NSM	rep 2	serotonine	1526	1010	177	2,79984E-96	3,01857E-96
AVK	rep 3	NA	1526	1803	230	8,11232E-87	8,6783E-87
OLQ	rep 2	glutamate	1526	915	160	1,42889E-86	1,51682E-86
I5	rep 1	glutamate	1526	799	149	4,88922E-77	5,15047E-77
I5	rep 2	glutamate	1526	805	150	1,05916E-76	1,1073E-76
RIS	rep 1	GABA	1526	1004	160	2,86281E-68	2,97043E-68
PHA	rep 1	glutamate	1526	813	133	5,02634E-67	5,17638E-67
PHA	rep 2	glutamate	1526	821	128	3,25781E-66	3,33021E-66
I5	rep 3	glutamate	1526	833	146	7,19841E-64	7,30427E-64
I5	rep 4	glutamate	1526	814	145	5,31123E-63	5,34999E-63
RIS	rep 2	GABA	1526	961	152	2,68747E-62	2,68747E-62

*: Genes expressed by the indicated neurons according to Hammarlund et al., Neuron 2018
p-values were obtained by two-sided Fischer’s exact test and corrected for multiple testing by the FDR method.

Table S19 - Lifespan analyses of Figure S5c

strain	RNAi	n_animals	median survival time [days]	% change vs control RNAi	pvalue (logrank test)
eri-1	control (L4440)	156	18,88		
eri-1	cfi-1#1	94	16,16	-14,4	6,83341E-07
eri-1	cfi-1#2	106	16,07	-14,9	2,61847E-07
eri-1	egl-5#1	137	19,08	1,0	0,331274436
eri-1	egl-5#2	133	19,64	4,0	0,014052493
eri-1	mab-5	93	18,58	-1,6	0,233717488
eri-1	unc-3#1	158	20,67	9,4	0,007972235
eri-1	unc-3#2	147	18,74	-0,8	0,896634359
eri-1	unc-4#1	137	19,69	4,2	0,358670598
eri-1	unc-4#2	141	18,96	0,4	0,429237623
daf-2; eri-1	control (L4440)	263	44,29		
daf-2; eri-1	cfi-1#1	139	40,94	-7,6	0,000168149
daf-2; eri-1	cfi-1#2	133	42,79	-3,4	0,047888695
daf-2; eri-1	egl-5#1	181	42,89	-3,2	0,160773653
daf-2; eri-1	egl-5#2	196	42,50	-4,0	0,255313518
daf-2; eri-1	mab-5	94	42,18	-4,8	0,000114041
daf-2; eri-1	unc-3#1	189	40,52	-8,5	6,06181E-08
daf-2; eri-1	unc-3#2	181	42,33	-4,4	0,030886507
daf-2; eri-1	unc-4#1	192	42,88	-3,2	0,093448812
daf-2; eri-1	unc-4#2	200	41,64	-6,0	0,016738897

Table S20 - Lifespan analyses of Figures 4c-d

experiment	strain	RNAi	n_animals	median survival time [days]	% change vs control RNAi	pvalue (logrank test)
displayed in figure	daf-2	control (L4440)	106	50,09090909		
displayed in figure	daf-2	lin-39	92	26,2	-47,7	2,13879E-32
displayed in figure	daf-2; unc-17 (low ACh signaling)	control (L4440)	163	57,70076726		
displayed in figure	daf-2; unc-17 (low ACh signaling)	lin-39	195	45,57142857	-21,0	3,1347E-23
displayed in figure	daf-2; unc-25 (low GABA signaling)	control (L4440)	156	43,63299663		
displayed in figure	daf-2; unc-25 (low GABA signaling)	lin-39	143	22,47619048	-48,5	1,34708E-46
replication	daf-2	control (L4440)	93	49,23809524		
replication	daf-2	lin-39	161	24,5625	-50,1	1,06859E-37
replication	daf-2; unc-17 (low ACh signaling)	control (L4440)	335	60,1875		
replication	daf-2; unc-17 (low ACh signaling)	lin-39	358	38,33333333	-36,3	2,73366E-11
replication	daf-2; unc-25 (low GABA signaling)	control (L4440)	160	46,54545455		
replication	daf-2; unc-25 (low GABA signaling)	lin-39	221	18,96547619	-59,3	2,34739E-73

Table S21 - Lifespan analysis of Figure 4e-g

Figure 4e

experiment	strain	RNAi	n_animals	median survival time [days]	% change vs control RNAi	pvalue (logrank test)
displayed in figure	daf-2; daf-16	control (L4440)	171	12,20		
displayed in figure	daf-2; daf-16	lin-39	193	11,40	-6,6	0,023502899
displayed in figure	daf-2	control (L4440)	133	41,93		
displayed in figure	daf-2	lin-39	291	19,48	-53,5	1,7347E-71
replication	daf-2; daf-16	control (L4440)	193	10,90		
replication	daf-2; daf-16	lin-39	210	11,18	2,6	0,407158272

Figure 4f-g

strain	RNAi	n_animals	median survival time [days]	% change vs control RNAi	pvalue (logrank test)
eri-1 males	control (L4440)	20	17,44		
eri-1 males	daf-16	10	9,50	-45,5	3,54377E-09
eri-1 males	lin-39	17	17,38	-0,4	0,897709928
daf-2; eri-1 males	control (L4440)	66	47,75		
daf-2; eri-1 males	daf-16	55	10,50	-78,0	1,59295E-33
daf-2; eri-1 males	lin-39	69	50,67	6,1	0,925387029

Table S22 - DARs affected by daf-16 RNAi and their overlap with DARs affected by lin-39 RNAi under reduced IIS

comparison #1*	comparison #2*	n_comparison #1	n_comparison #2	overlap	FDR	significant enrichment/depletion§	Log2 Odds Ratio
daf-2(e1370); daf-16(RNAi) < daf-2(e1370)	daf-2(e1370); daf-16(RNAi) < daf-2(e1370)	5711	5711	5711	0		
daf-2(e1370); daf-16(RNAi) < daf-2(e1370)	daf-2(e1370); lin-39(RNAi) < daf-2(e1370)	5711	303	200	3,115E-116	enriched	4,282730672
daf-2(e1370); daf-16(RNAi) < daf-2(e1370)	daf-2(e1370); lin-39(RNAi) > daf-2(e1370)	5711	331	11	2,22207E-06	depleted	-1,801695055
daf-2(e1370); daf-16(RNAi) > daf-2(e1370)	daf-2(e1370); daf-16(RNAi) > daf-2(e1370)	2261	2261	2261	0		
daf-2(e1370); daf-16(RNAi) > daf-2(e1370)	daf-2(e1370); lin-39(RNAi) < daf-2(e1370)	2261	303	2	0,000316117	depleted	-2,890005437
daf-2(e1370); daf-16(RNAi) > daf-2(e1370)	daf-2(e1370); lin-39(RNAi) > daf-2(e1370)	2261	331	35	9,92663E-07	enriched	1,492245218
daf-2(e1370); lin-39(RNAi) < daf-2(e1370)	daf-2(e1370); daf-16(RNAi) < daf-2(e1370)	303	5711	198	2,92534E-85	enriched	3,413004927
daf-2(e1370); lin-39(RNAi) < daf-2(e1370)	daf-2(e1370); daf-16(RNAi) > daf-2(e1370)	303	2261	2	0,004692862	depleted	-2,505420825
daf-2(e1370); lin-39(RNAi) < daf-2(e1370)	daf-2(e1370); lin-39(RNAi) < daf-2(e1370)	303	303	303	0		
daf-2(e1370); lin-39(RNAi) > daf-2(e1370)	daf-2(e1370); daf-16(RNAi) < daf-2(e1370)	331	5711	11	1,27627E-11	depleted	-2,428512443
daf-2(e1370); lin-39(RNAi) > daf-2(e1370)	daf-2(e1370); daf-16(RNAi) > daf-2(e1370)	331	2261	35	9,2349E-08	enriched	1,691350633
daf-2(e1370); lin-39(RNAi) > daf-2(e1370)	daf-2(e1370); lin-39(RNAi) > daf-2(e1370)	331	331	331	0		

*: X < Y indicates DARs which are open in Y and closed in X. X > Y indicates DARs which are closed in Y and open in X.

§: significance cutoff is FDR<0.05.

FDR: False Discovery Rate

WT: wild-type (N2)

Table S23 - Overlap of DARs opened by DAF-16 with different chromatin regions, as determined by ATAC-seq and data from Ho et al.

chromatin regions[#]	n_DARs	n_genomic regions	overlap	FDR	significant enrichment/depletion[§]	Log2 Odds Ratio
Enhancers	5711	6496	3328	0.00e+00	enrichment	2,538290351
Promoters	5711	2661	1560	0.00e+00	enrichment	2,547167919
3'-UTRs	5711	7337	292	0.00e+00	depletion	-2,907921643
PcG-repressed	5711	2033	139	1.69e-107	depletion	-2,207430228
Low signal	5711	6523	595	8.59e-90	depletion	-1,197404918
Heterochromatin	5711	2654	205	6.62e-85	depletion	-1,738648135
Gene bodies	5711	965	180	7.00e-22	depletion	-0,998177225
5'-UTRs	5711	1667	445	4.20e-01		0,061686141

[#]: Chromatin regions were taken from Ho et al., Nature 2014. Naming of some chromatin regions was slightly adapted to improve readability.

[§]: significance cutoff is FDR<0.05.

FDR: False Discovery Rate

PcG-repressed: Polycomb Group protein-repressed

Table S24 - Overlap of DARs that require both LIN-39 and DAF-16 for their opening with different chromatin regions, as determined by ATAC-seq and data from Ho et al.

chromatin regions[#]	n_DARs	n_genomic regions	overlap	FDR	significant enrichment/depletion[§]	Log2 Odds Ratio
Enhancers	200	6496	142	9.72e-33	enrichment	2,521865628
3'-UTRs	200	7337	7	1.03e-14	depletion	-3,067453565
Gene bodies	200	965	1	5.97e-04	depletion	-3,468571697
PcG-repressed	200	2033	7	4.07e-03	depletion	-1,46513954
Promoters	200	2661	15	7.55e-02		-0,68905239
Low signal	200	6523	27	1.16e-01		-0,492799408
5'-UTRs	200	1667	12	5.01e-01		-0,361749859
Heterochromatin	200	2654	15	5.37e-01		-0,307557164

[#]: Chromatin regions were taken from Ho et al., Nature 2014. Naming of some chromatin regions was slightly adapted to improve readability.

[§]: significance cutoff is FDR<0.05.

FDR: False Discovery Rate

PcG-repressed: Polycomb Group protein-repressed

Table S25 - Functional enrichments (GO-BP) of genes closest to DARs that require both LIN-39 and DAF-16 for their opening under reduced IIS

Only the first 30 terms are shown.

GO-BP term	n_genes	FDR	significant enrichment/depletion[§]	Log2 Odds Ratio
cell-cell adhesion	7	3.89e-05	enrichment	4,459221185
cell morphogenesis	11	3.89e-05	enrichment	3,221171197
cell morphogenesis involved in differentiation	10	5.64e-05	enrichment	3,290766846
tissue development	9	2.83e-04	enrichment	3,171570723
plasma membrane bounded cell projection organization	11	4.43e-04	enrichment	2,696704337
microtubule-based process	10	9.86e-04	enrichment	2,658404404
embryonic morphogenesis	8	9.86e-04	enrichment	3,045540999
cell morphogenesis involved in neuron differentiation	8	1.05e-03	enrichment	3,004868208
neuron projection morphogenesis	8	1.06e-03	enrichment	2,949750072
plasma membrane bounded cell projection morphogenesis	8	1.06e-03	enrichment	2,949750072
cell projection morphogenesis	8	1.06e-03	enrichment	2,934373422
cell part morphogenesis	8	1.12e-03	enrichment	2,904092125
nervous system development	11	1.20e-03	enrichment	2,375130787
animal organ development	11	1.48e-03	enrichment	2,327246384
neuron projection development	8	2.59e-03	enrichment	2,67439711
positive regulation of macromolecule biosynthetic process	9	2.87e-03	enrichment	2,457515619
positive regulation of biosynthetic process	9	3.02e-03	enrichment	2,423512294
positive regulation of cellular biosynthetic process	9	3.02e-03	enrichment	2,423512294
cell migration	7	3.08e-03	enrichment	2,808399185
cytoskeleton organization	10	4.63e-03	enrichment	2,178530769
localization of cell	7	4.63e-03	enrichment	2,68331076
regulation of anatomical structure morphogenesis	6	5.01e-03	enrichment	2,927440249
microtubule cytoskeleton organization	7	9.27e-03	enrichment	2,486582811
embryo development ending in birth or egg hatching	8	1.05e-02	enrichment	2,254031613
microtubule cytoskeleton organization involved in mitosis	5	1.06e-02	enrichment	3,016545733
positive regulation of nitrogen compound metabolic process	10	1.19e-02	enrichment	1,944314697
cytoskeleton-dependent intracellular transport	4	1.23e-02	enrichment	3,429790378
positive regulation of developmental process	7	1.85e-02	enrichment	2,254638368
regulation of multicellular organismal development	6	1.85e-02	enrichment	2,471599573
mitotic cell cycle process	7	1.87e-02	enrichment	2,193548125

[§]: significance cutoff is FDR<0.05.
FDR: False Discovery Rate

Table S26 - Functional enrichments (GO-BP) of genes differentially expressed under reduced IIS in VC neurons (based on Ange et al., Cell Genomics 2024)

DEGs upregulated in daf-2 vs wild-type
Only the first 30 terms are shown.

GO-BP term	n_genes	FDR	significant enrichment/depletion§
neuropeptide signaling pathway	27	1,61E-12	enrichment
detection of external stimulus	10	2,09E-05	enrichment
detection of abiotic stimulus	10	2,09E-05	enrichment
negative regulation of behavior	7	0,000535	enrichment
negative regulation of multicellular organismal process	14	0,001770	enrichment
cellular response to oxygen-containing compound	14	0,001770	enrichment
detection of mechanical stimulus involved in sensory perception	5	0,002238	enrichment
detection of mechanical stimulus involved in sensory perception of touch	5	0,002238	enrichment
sensory perception of mechanical stimulus	6	0,004529	enrichment
sensory perception of touch	5	0,005151	enrichment
detection of mechanical stimulus	5	0,005151	enrichment
dopamine transport	5	0,005374	enrichment
catecholamine transport	5	0,005374	enrichment
regulation of membrane potential	15	0,005374	enrichment
chemical synaptic transmission	20	0,005374	enrichment
anterograde trans-synaptic signaling	20	0,005374	enrichment
trans-synaptic signaling	20	0,005374	enrichment
amine transport	6	0,005374	enrichment
glutamate receptor signaling pathway	5	0,005374	enrichment
response to oxygen-containing compound	15	0,005374	enrichment
synaptic signaling	20	0,006240	enrichment
ionotropic glutamate receptor signaling pathway	4	0,007551	enrichment
monoamine transport	5	0,007551	enrichment
response to inorganic substance	12	0,007551	enrichment
defense response to fungus	6	0,007551	enrichment
response to fungus	6	0,008727	enrichment
ligand-gated ion channel signaling pathway	4	0,009216	enrichment
mating behavior	7	0,009562	enrichment
locomotory behavior	9	0,010067	enrichment
phototransduction	4	0,011337	enrichment

DEGs downregulated in daf-2 vs wild-type
Only the first 30 terms are shown.

GO-BP term	n_genes	FDR	significant enrichment/depletion§
neuropeptide signaling pathway	47	1,49E-21	enrichment
locomotion	50	0,000428	enrichment
turning behavior involved in mating	7	0,000428	enrichment
negative regulation of behavior	9	0,000639	enrichment
regulation of male mating behavior	7	0,001008	enrichment
male mating behavior	11	0,001145	enrichment
response to abiotic stimulus	35	0,001145	enrichment
muscle system process	15	0,001145	enrichment
muscle contraction	15	0,001145	enrichment
collagen and cuticulin-based cuticle development	12	0,001154	enrichment
nervous system development	45	0,002756	enrichment
regulation of behavior	24	0,002895	enrichment
mating behavior	11	0,003288	enrichment
neuron differentiation	35	0,004084	enrichment
cuticle development	12	0,004084	enrichment
gas transport	8	0,005800	enrichment
oxygen transport	8	0,005800	enrichment
generation of neurons	36	0,005932	enrichment
regulation of system process	14	0,008199	enrichment
neurogenesis	36	0,014698	enrichment
molting cycle, collagen and cuticulin-based cuticle	16	0,015167	enrichment
rhythmic process	6	0,021360	enrichment
molting cycle	16	0,021360	enrichment
defense response to Gram-negative bacterium	17	0,021360	enrichment
cell junction organization	17	0,021360	enrichment
cell projection organization	35	0,021360	enrichment
plasma membrane bounded cell projection organization	34	0,021360	enrichment
cell junction assembly	12	0,021360	enrichment
regulation of monoatomic ion transmembrane transport	10	0,021360	enrichment
response to oxygen levels	10	0,021360	enrichment

§: significance cutoff is FDR<0.05.
FDR: False Discovery Rate

Table S27 - *C. elegans* strains used in this study

Strain	Genotype	Source	Used in figure(s)	Labels used in figure(s) to refer to this strain
SS104	*glp-4(bn2) I*	Caenorhabditis Genetics Center (CGC)	1, S1	*glp-4, day1, day3, or day7*
GR1373	*eri-1(mg366) IV*	Riedel et al., Nature Cell Biology 2013	1, 2c-d, 3a, 4f-g, S1, S2, S3	wild-type, WT, or *eri-1*
GR1899	*daf-2(e1370) III; eri-1(mg366) IV*	Riedel et al., Nature Cell Biology 2013	1, 2c-d, 3a, 4e-g, S1, S2, S3, S5c, S8	*daf-2 or daf-2; eri-1*
N2	wild-type	Caenorhabditis Genetics Center (CGC)	2e	wild-type
OP18	*unc-119(ed3) III; wgIs18 [lin-39::TY1::EGFP::3xFLAG + unc-119(+)]*	Caenorhabditis Genetics Center (CGC)	2e, S7	*lin-39 OE or lin-39::GFP*
IJ415	*daf-2(e1370) III; rde-1(ne219) V; kzIs9[pKK1260(lin-26p::nls::GFP) + pKK1253(lin-26p::rde-1) + pRF6(rol-6(su1006)]*	Son et al., Nature Communications 2017	3a	Hypodermis
IJ416	*daf-2(e1370) III; rde-1(ne219) V; kzIs20[pDM#715(hlh-1p::rde-1) + pTG95(sur-5p::nls::GFP)]*	Son et al., Nature Communications 2017	3a	Muscle
IJ417	*daf-2(e1370) III; rde-1(ne213) V; kbIs7[nhx-2p::rde-1 + rol-6(su1006)]*	Son et al., Nature Communications 2017	3a	Intestine
RIE521	*daf-2(e1370) III; sid-1(pk3321) V; uIs69[pCFJ90 (myo-2p::mCherry) + unc-119p::sid-1] V*	This study, derived from Zullo et al., Nature 2019	3a-c, S4	Neurons or RNAi in neurons
CB1370	*daf-2(e1370) III*	Caenorhabditis Genetics Center (CGC)	4c-d	*daf-2*
RIE578	*daf-2(e1370) III; unc-25(e156) III*	This study	4c	low GABA
RIE576	*daf-2(e1370) III; unc-17(e245) IV*	This study	4d	low ACh
GR1901	*daf-16(mgDf47) I; daf-2(e1370) III; eri-1(mg366) IV*	Riedel et al., Nature Cell Biology 2013	4e, S8	*daf-2; daf-16; eri-1*
LX929	*vsIs48[unc-17p::unc-17::GFP]*	Caenorhabditis Genetics Center (CGC)	S6a	*unc-17::GFP*
RIE665	*daf-2(e1370) III; vsIs48[unc-17p::unc-17::GFP]*	This study	S6b	*daf-2; unc-17::GFP*
RIE502	*daf-2(e1370) III; unc-119(ed3) III; wgIs18 [lin-39::TY1::EGFP::3xFLAG + unc-119(+)]*	This study	S7	*daf-2; lin-39::GFP*

Supplementary references

1. Ho, J. W. K. *et al.* Comparative analysis of metazoan chromatin organization. *Nature* **512**, 449–452 (2014).

2. Gerstein, M. B. *et al.* Integrative Analysis of the Caenorhabditis elegans Genome by the modENCODE Project. *Science* **330**, 1775–1787 (2010).

3. Wang, X. *et al.* Ageing induces tissue - specific transcriptomic changes in Caenorhabditis elegans. *EMBO J.* **41**, e109633 (2022).

4. Hammarlund, M., Hobert, O., Miller, D. M. & Sestan, N. The CeNGEN Project: The Complete Gene Expression Map of an Entire Nervous System. *Neuron* **99**, 430–433 (2018).

5. Taylor, S. R. *et al.* Molecular topography of an entire nervous system. *Cell* **184**, 4329-4347.e23 (2021).
